# The *HHIP-AS1* lncRNA promotes tumorigenicity through stabilization of dynein complex 1 in human SHH-driven tumors

**DOI:** 10.1038/s41467-022-31574-z

**Published:** 2022-07-13

**Authors:** Jasmin Bartl, Marco Zanini, Flavia Bernardi, Antoine Forget, Lena Blümel, Julie Talbot, Daniel Picard, Nan Qin, Gabriele Cancila, Qingsong Gao, Soumav Nath, Idriss Mahoungou Koumba, Marietta Wolter, François Kuonen, Maike Langini, Thomas Beez, Christopher Munoz, David Pauck, Viktoria Marquardt, Hua Yu, Judith Souphron, Mascha Korsch, Christina Mölders, Daniel Berger, Sarah Göbbels, Frauke-Dorothee Meyer, Björn Scheffler, Barak Rotblat, Sven Diederichs, Vijay Ramaswamy, Hiromishi Suzuki, Anthony Oro, Kai Stühler, Anja Stefanski, Ute Fischer, Gabriel Leprivier, Dieter Willbold, Gerhard Steger, Alexander Buell, Marcel Kool, Peter Lichter, Stefan M. Pfister, Paul A. Northcott, Michael D. Taylor, Arndt Borkhardt, Guido Reifenberger, Olivier Ayrault, Marc Remke

**Affiliations:** 1 Division of Pediatric Neuro-Oncogenomics, German Cancer Research Center (DKFZ), Heidelberg, Germany, and German Cancer Consortium (DKTK), partner site Essen/Düsseldorf, Düsseldorf, Germany; 2 Department of Pediatric Oncology, Hematology, and Clinical Immunology, Medical Faculty, Heinrich Heine University, Düsseldorf, Germany, and DKTK, partner site Essen/Düsseldorf, Düsseldorf, Germany; 3 Institute of Neuropathology, Medical Faculty, Heinrich Heine University, Düsseldorf, Germany, DKTK, partner site Essen/Düsseldorf, Düsseldorf, Germany; 4 Group for Interdisciplinary Neurobiology and Immunology-INI-research, Institute of Zoology University of Hamburg, Hamburg, Germany; 5 Institut Curie, PSL Research University, CNRS UMR, INSERM, Orsay, France; 6 Université Paris Sud, Université Paris-Saclay, CNRS UMR, INSERM U, Orsay, France; 7 St Jude Children’s Research Hospital, Memphis, TN USA; 8 Institut für Physikalische Biologie and Biological-Medical Research Center (BMFZ), Heinrich Heine University, Düsseldorf, Germany; 9 IBI- (Strukturbiochemie) and JuStruct, Forschungszentrum Jülich, Jülich, Germany; 10 Program in Epithelial Biology, Stanford University School of Medicine, Stanford, CA USA; 11 Department of Dermatology and Venereology, Hôpital de Beaumont, Lausanne University Hospital Center, CH- Lausanne, Lausanne, Switzerland; 12 Institute for Molecular Medicine, Proteome Research, Medical Faculty, Heinrich Heine University, Düsseldorf, Germany; 13 Department of Neurosurgery, Medical Faculty, Heinrich Heine University, Düsseldorf, Germany; 14 DKFZ Division of Translational Neurooncology at the West German Cancer Center (WTZ), DKTK, partner site University Hospital Essen, Düsseldorf, Germany; 15 Department of Life Sciences, Ben-Gurion University of the Negev, Beer Sheva, Israel; 16 The National Institute for Biotechnology in the Negev, Beer Sheva, Israel; 17 Division of Cancer Research, Department of Thoracic Surgery, Medical Center-University of Freiburg, Faculty of Medicine, University of Freiburg, DKTK, partner site Freiburg, Freiburg i.Br, Germany; 18 Division of RNA Biology & Cancer, DKFZ, Heidelberg, Germany; 19 Developmental and Stem Cell Biology Program, The Hospital for Sick Children, Toronto, Ontario Canada; 20 Division of Haematology/Oncology, Department of Pediatrics, The Hospital for Sick Children, Toronto, Ontario Canada; 21 Department of Dermatology, Stanford University, Stanford, CA USA; 22 Molecular Proteomics Laboratory (MPL), BMFZ, Heinrich Heine University, Düsseldorf, Germany; 23 Department of Biotechnology and Biomedicine, Technical University of Denmark, Kongens Lyngby, Denmark; 24 Hopp Children´s Cancer Center (KiTZ), Heidelberg, Germany; 25 Division of Pediatric Neurooncology, German Cancer Research Center (DKFZ) and German Cancer Consortium (DKTK), Heidelberg, Germany; 26 Princess Máxima Center for Pediatric Oncology, Utrecht, the Netherlands; 27 Division of Molecular Genetics, German Cancer Research Center (DKFZ), German Cancer Consortium (DKTK), and National Center for Tumor Diseases (NCT), Heidelberg, Germany; 28 Department of Pediatric Hematology and Oncology, Heidelberg University Hospital, Heidelberg, Germany; 29 The Arthur and Sonia Labatt Brain Tumour Research Centre, The Hospital for Sick Children, Toronto, Ontario Canada; 30 Department of Laboratory Medicine and Pathobiology, University of Toronto, Toronto, Ontario Canada; 31 Division of Neurosurgery, The Hospital for Sick Children, Toronto, Ontario Canada

**Keywords:** Mitotic spindle, Long non-coding RNAs, Paediatric cancer

## Abstract

Most lncRNAs display species-specific expression patterns suggesting that animal models of cancer may only incompletely recapitulate the regulatory crosstalk between lncRNAs and oncogenic pathways in humans. Among these pathways, Sonic Hedgehog (SHH) signaling is aberrantly activated in several human cancer entities. We unravel that aberrant expression of the primate-specific lncRNA *HedgeHog Interacting Protein-AntiSense 1* (*HHIP-AS1*) is a hallmark of SHH-driven tumors including medulloblastoma and atypical teratoid/rhabdoid tumors. *HHIP-AS1* is actively transcribed from a bidirectional promoter shared with SHH regulator *HHIP*. Knockdown of *HHIP-AS1* induces mitotic spindle deregulation impairing tumorigenicity in vitro and in vivo. Mechanistically, *HHIP-AS1* binds directly to the mRNA of *cytoplasmic dynein 1 intermediate chain 2* (*DYNC1I2*) and attenuates its degradation by hsa-miR-425-5p. We uncover that neither *HHIP-AS1* nor the corresponding regulatory element in *DYNC1I2* are evolutionary conserved in mice. Taken together, we discover an lncRNA-mediated mechanism that enables the pro-mitotic effects of SHH pathway activation in human tumors.

## Abstract (web summary)

Long non-coding RNAs (lncRNAs) can contribute to cancers that are driven by Sonic hedgehog (SHH) signaling. Here the authors report that lncRNA *HHIP-AS1* stabilises the mRNA of dynein complex 1, thereby, promoting the pro-mitotic effects of SHH-driven tumors.

## Introduction

Sonic hedgehog (SHH) signaling plays a pivotal role in promoting oncogenesis, tumor growth and progression^1^. It is aberrantly activated in various common cancers in adults, including basal cell carcinoma (BCC)^2^, but also in pediatric neoplasms, including rhabdomyosarcoma^3^ and brain tumors such as medulloblastoma (MB) and atypical teratoid/rhabdoid tumors (ATRT)^4–8^. Pediatric brain tumors, like MB and ATRT, are the most common solid malignancies of childhood and the leading cause of cancer-related death in children^9^. Both entities are highly heterogeneous and can be segregated into distinct subgroups by virtue of their divergent molecular characteristics. Notably, such classification, which is primarily based on intergroup differences detected at multi-omics level, is of clinical utility, as it correlates with specific and distinct clinico-pathological and prognostic patterns^10–12^. Specifically, MB comprises four main subgroups, designated as Wingless (WNT), SHH, Group 3 and Group 4^13–16^. WNT and SHH MBs are named according to the signaling pathways that drive their formation and progression^17,18^. Conversely, the other two MB subgroups are less defined in their molecular etiology^19,20^, although Group 3 MB recurrently displays *MYC* amplification and/or overexpression^21–24^, while Group 4 MB commonly show activation of receptor tyrosine kinase signaling through aberrant expression of ERBB4 and the phosphorylated form of the tyrosine–protein kinase SRC^25^. In the case of ATRT, three subgroups have been defined, namely tyrosinase (TYR), MYC and SHH, according to the distinctive overexpression of *TYR* or *MYC* genes, or the activation of SHH signaling^4,6,26^.

Targeting the SHH signaling to extinguish its mitogenic effects in SHH MB has shown efficacy in pre-clinical animal models and in humans^27^. However, the clinical use of SHH inhibitors, such as the G protein-coupled receptor smoothened (SMO) antagonist Vismodegib (GDC-0449), is limited due to toxicity or emergence of drug resistance in children with SHH MB to date^28,29^. While mutation and altered expression of several protein-coding genes are well-established drivers of SHH-dependent tumorigenesis, the precise impact of the non-protein coding genome remains largely unexplored. In particular, investigations of the specific role of long non-coding RNAs (lncRNAs) in SHH-driven malignancies are just emerging as an important research field^30,31^. Most research efforts have so far focused on well-defined and comprehensive murine models of SHH-driven tumors^32,33^. However, these models fail to offer a faithful representation of the human lncRNAs landscape, because lncRNAs show only poor conservation across species^34^. Nevertheless, lncRNAs are known to play essential roles in all aspects of cell biology^35–37^ and the regulatory mechanisms orchestrated by lncRNAs are diverse including direct binding to chromatin^38^, proteins^39^ or regulating microRNAs^40^. Conversely, the role of non-coding RNAs in the pathogenesis and disease stratification of ATRT has not been elucidated so far, and non-coding RNA classes also remain poorly characterized in MB.

Here, we explore the role of lncRNAs specifically in SHH-driven tumors with the aim to decipher the impact in SHH signaling regulation and function in these tumors. We show that aberrant expression of the lncRNA *Hedgehog Interacting Protein Antisense 1* (*HHIP-AS1*) is a hallmark of human SHH-driven tumors including MB and ATRT. *HHIP-AS1* is actively transcribed from a SHH-responsive bidirectional promoter shared with the SHH signaling intermediate *HHIP*. *HHIP-AS1* knockdown leads to reduced tumor growth in SHH-driven tumors in vitro and in vivo by decreasing cell proliferation and inducing mitotic spindle deregulation. Mechanistically, we show that *HHIP-AS1* binds to and stabilizes the mRNA of *cytoplasmic dynein 1 intermediate chain 2* (*DYNC1I2*), a component of the cytoplasmic dynein 1 complex, by preventing hsa-miR-425-5p-mediated degradation. We uncover that neither *HHIP-AS1* nor the corresponding regulatory element in *DYNC1I2* are evolutionary conserved in mice demonstrating the power of cross-entity transcriptome-wide comparisons to identify regulatory lncRNA circuitries in human cancer. In all, we discover a lncRNA-dependent blockage of endogenous RNA mechanism as an additional layer of epigenetic regulation mediated by *HHIP-AS1* as a human-specific target gene in SHH-dependent cell progression.

## Results

### Overexpression of the long non-coding RNA *HHIP-AS1* is a hallmark of human SHH-driven tumors

**Fig. 1:**
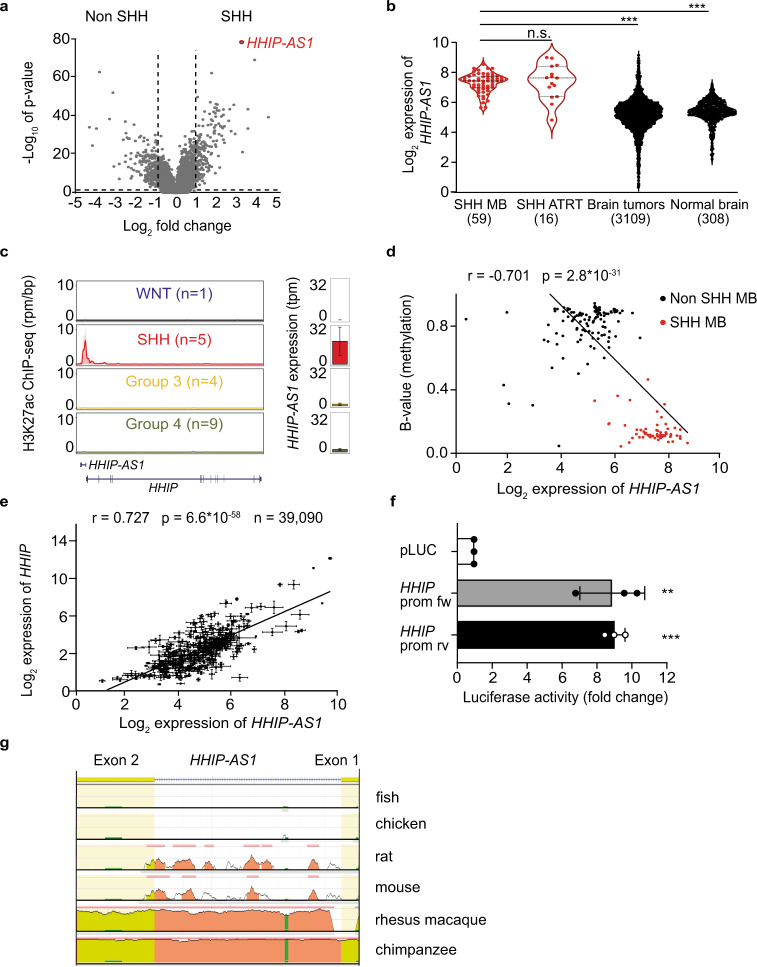
The long non-coding RNA *HHIP-AS1* is a hallmark of human SHH-driven tumors. **a** Long non-coding RNA (lncRNA) expression profiles in SHH-driven medulloblastoma (MB; right side) *versus* non SHH-driven MB (left side). The volcano plot illustrates the distribution of statistical significance (*y*-axis) and relative expression level (*x*-axis) for the lncRNAs profile. The red dot indicates *HHIP-AS1* (*HedgeHog Interacting Protein-Anti-Sense 1*). Statistical analysis was performed using one-way ANOVA with post-hoc Tukey HSD; ***p < 0.001. **b** Violin plots display the expression level of *HHIP-AS1* according to an integrative transcriptomic analysis of 3492 samples from neoplastic brain tissues with SHH activation (SHH MB and atypical teratoid/rhabdoid tumors (ATRT)) or without commonly reported SHH activation (brain tumors) and normal brain without tumor. Statistical analysis was performed using Kruskal–Wallis Test with Dunn’s Multiple Comparison Test; ***p < 0.001. Red dots = SHH-driven entities; black dots = non SHH-driven tumors and control tissue. **c** H3K27ac ChIP-sequencing profile on *HHIP-AS1* and *HHIP* loci in the four MB subgroups^64^. Bar graph indicates the expression level of *HHIP-AS1* in the corresponding MB subgroups. Error bars represent ± SEM. **d** Scatter plot representing the degree of DNA methylation (B value) of the potential promoter region in relation to *HHIP-AS1* expression levels in SHH MB (red dots) and other MB subgroups (black dots). Statistics were done by Pearson correlation. **e** Scatter plot displaying expression correlation of *HHIP* and *HHIP-AS1* across datasets (*n* = 351). Mean expressions of both transcripts from 39,090 samples were analyzed in their respective datasets and plotted with error bars representing the SEM for both genes. Statistics were done by Pearson correlation. **f** The bar graph indicates the relative luciferase activity of empty luciferase vector (pLUC) or pLUC containing the cloned *HHIP* promoter sequence orientation (fw = forward, rv = reversed). The results are presented as the mean ± SD of three independent experiments. Student´s two-sided *t*-test; **p < 0.01, ***p < 0.001. **g** Identification of evolutionarily conserved regions corresponding to critical regulatory elements in large (>1 Mb), highly conserved gene desert regions flanking the human *HHIP-AS1* gene located at chromosome 4q31.21 with two exons. Source data and exact *p*-values are provided as a “Source Data file”.

To discover global SHH-dependent gene expression patterns in cancer, we first determined differentially expressed transcripts in RNA sequencing data by comparing SHH MB (*n* = 58) to non SHH MB subgroup samples (*n* = 164) (Platform, R2^41^). Our approach confirmed several known protein-coding SHH mediators including *GLI1, GLI2, HHIP* and *Atonal BHLH Transcription Factor 1*, and revealed lncRNAs deregulated in SHH MB (Fig. 1a, Supplementary Data file 1). Among these candidates, we identified *HHIP-AS1* as the most specifically overexpressed lncRNA in SHH MB (Fig. 1a, Supplementary Data file 1) without protein-coding potential (Fig. S1a). We next investigated whether *HHIP-AS1* was similarly overexpressed in other tumor entities with aberrant activation of the SHH signaling. Comparative expression analysis confirmed specific *HHIP-AS1* overexpression in SHH-driven entities including ATRT (Fig. 1b), cutaneous BCC (Fig. S1b) and rhabdomyosarcoma (Fig. S1c) compared to normal and cancerous control tissues. Consistent with its overexpression, we next revealed active transcription of *HHIP-AS1* from a hypomethylated promoter shared with *HHIP*, a well-established regulator of the SHH pathway and whose genomic localization shows a head-to-head orientation with the *HHIP-AS1* locus, exclusively in SHH MB using both H3K27ac ChIP-sequencing (Fig. 1c) and high-resolution DNA methylation data (Fig. 1d and S1d). Transcriptome-wide correlation analyses consistently revealed that *HHIP* and *HHIP-AS1* are co-expressed in MB (*n* = 167, *r* = 0.990, *p* < 0.001, Fig. S1e) and in ATRT (*n* = 49, *r* = 0.836, *p* < 0.001, Fig. S1f) cohorts. Furthermore, we could reveal that the two genes exhibited remarkably high and significant correlative co-expression scores in multiple expression datasets, including other cancer entities and control tissues (*n* = 39,090; *p* < 0.001, Fig. 1e). These observations prompted us to hypothesize that *HHIP-AS1* and *HHIP* may indeed share a unique bidirectional promoter. Therefore, we performed a luciferase reporter assay and revealed the existence of forward and reverse activity of the promoter region in vitro (Fig. 1f), suggesting that promoter hypomethylation may equally drive overexpression of both *HHIP-AS1* and *HHIP* transcripts in SHH-driven tumors.

Since *HHIP-AS1* was not previously described in murine models of SHH-driven tumors and lncRNAs are only partially conserved through species^34^, we investigated to which extent *HHIP-AS1* was conserved across vertebrates. Remarkably, genome-scale comparisons revealed that *HHIP-AS1* was only conserved in primates and not in rodents (Fig. 1g and S1g), explaining why this lncRNA has not been identified in animal models so far.

### *HHIP-AS1* is functionally required in human SHH-driven brain tumors

**Fig. 2:**
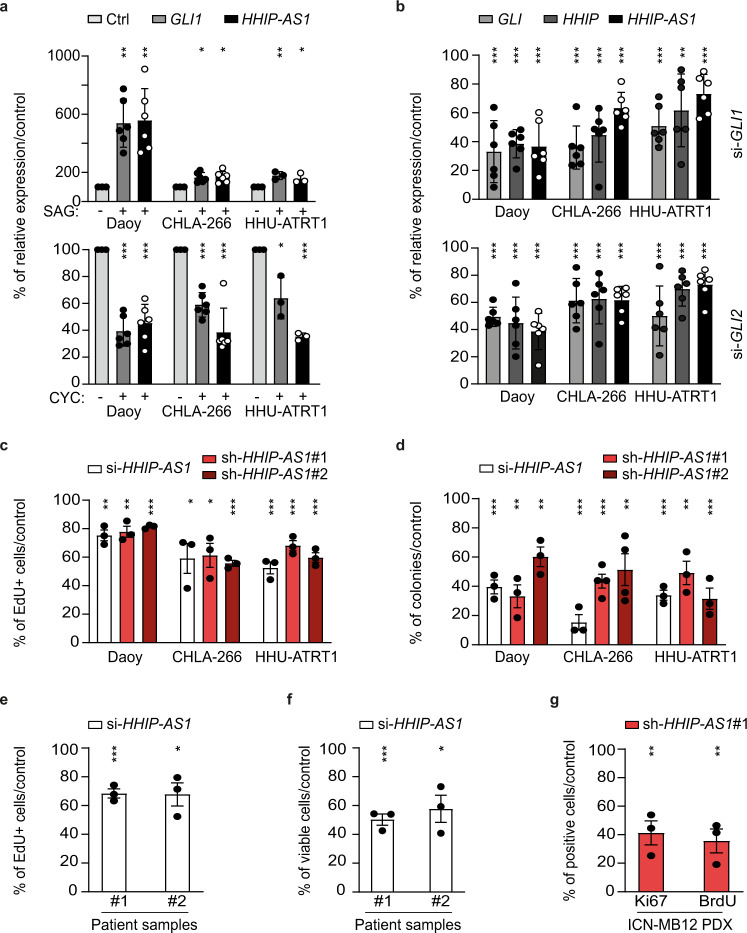
The long non-coding RNA *HHIP-AS1* is functionally required in human SHH-driven brain tumors. **a** The relative gene expression levels of *HHIP-AS1* and the SHH target gene *GLI1* were tested in tumor cell lines (Daoy and CHLA-266) and in primary tumor cell cultures (HHU-ATRT1) upon pharmacological activation (SAG, Smoothened agonist) or inhibition (CYC, cyclopamine) of the SHH pathway. **b** Relative gene expression levels of indicated genes as measured by qRT-PCR upon transient knockdown of *GLI1* and *GLI2* in the depicted cell models normalized to control (si-*negative*-POOL; gene expression of target genes were normalized to housekeeping genes: *HPRT*, *GUSB* and *PPIA*). **c** Proliferation rate of Daoy, CHLA-266 and HHU-ATRT1 was measured by EdU incorporation upon transient (si-*HHIP-AS1*) or stable *HHIP-AS1* knockdown normalized to control. **d** Self-renewal capacity of Daoy, CHLA-266 and HHU-ATRT1 was measured by colony formation assay upon transient (si-*HHIP-AS1*) or stable (sh-*HHIP-AS1*#1 and sh-*HHIP-AS1*#2) *HHIP-AS1* knockdown normalized to control. In panel **c** + **d** corresponding controls (either with si-*negative*-POOL or sh-*scr* transfected Daoy, CHLA-266 and HHU-ATRT1 cells) were set to 100% and levels of knockdowns were calculated accordingly. **e** Proliferation rate of primary SHH MB cultures derived from freshly resected tumors (*n* = 2 patients) measured by EdU incorporation upon transient knockdown of *HHIP-AS1* (si*-HHIP-AS1*) normalized to control (si-*negative*-POOL). **f** Cell viability of these primary SHH MB cultures derived from freshly resected tumors (*n* = 2 patients) measured by CellTiter-Glo upon transient knockdown of *HHIP-AS1* (si*-HHIP-AS1*) normalized to control (si-*negative*-POOL). **g** Proliferation rate of SHH MB PDX cells (ICN-MB12) determined by BrdU incorporation and Ki67 immunostaining after transient knockdown of *HHIP-AS1* (sh-*HHIP-AS1#1*) normalized to control. Bar graphs of panels **a** + **b** are presented as the mean ± SD, panels **c**–**g** are presented as the mean ± SEM of at least three independent experiments and corresponding controls were set to 100%. Student’s two-sided *t*-test; ***p < 0.001; **p < 0.01; *p < 0.05. Source data and exact *p*-values are provided as a “Source Data file”.

Since *HHIP-AS1* overexpression is a hallmark in SHH-driven tumors, we investigated whether *HHIP-AS1* expression was dependent on SHH signaling in tumor cells. Pharmacological activation of SMO receptor using SAG (SMO agonist) increased *HHIP-AS1* expression in two independent tumor cell lines, Daoy and CHLA-266, and one primary cell culture, HHU-ATRT1 (Fig. 2a) as well as in non-tumor cells with SHH activation, namely neuronal stem cells (NSC; Fig. S3a). Conversely, inhibition of SMO using cyclopamine (SMO antagonist) significantly reduced *HHIP-AS1* in all these cell models (Fig. 2a and S3a). Activation and inhibition of the SHH pathway were confirmed by quantification of GLI expression level through qRT-PCR (Fig. 2a and S3a) and immunoblotting (Fig. S2a–c). GLI proteins are known to function as regulators of the SHH transcriptional response, hence we next examined whether GLI could affect *HHIP-AS1* expression. Transient knockdown of *GLI1* and *GLI2* reduced *HHIP-AS1* and *HHIP* expression in all of the above-mentioned SHH-cancer cell models (Fig. 2b and S2), while on the other hand overexpression of GLI1 (Fig. S2d) resulted in increased expression of *HHIP* and *HHIP-AS1* (Fig. S3b), confirming *HHIP-AS1* as a target gene of SHH signaling. To evaluate the functional impact of *HHIP-AS1*, we silenced its expression in vitro using either siRNAs or shRNAs directed against this lncRNA. Strikingly, knockdown of *HHIP-AS1* (Fig. S3c) resulted in reduced proliferation, monitored by EdU incorporation (Fig. 2c), as well as reduced clonogenicity (Fig. 2d) of Daoy, CHLA-266 and HHU-ATRT1. In addition, cell viability, assessed through metabolic assay (CellTiter-Glo), was diminished in the SHH MB cell line Daoy and in ATRT cell line CHLA-266 upon *HHIP-AS1* silencing (Fig. S3d). We could verify these phenotypic changes after transient *HHIP-AS1* knockdown in additional SHH-driven cell models, namely CHLA-04 (ATRT), RH30 (rhabdomyosarcoma), NSC and UI226 (BCC, Fig. S3e). Importantly, we also confirmed that *HHIP-AS1* depletion reduced proliferation and viability in primary cultures from two SHH MB patients (Fig. 2e and f) and impaired proliferation of cultured cells of ICN-MB12 (SHH MB patient derived xenograft model; Fig. 2g). Notably, overexpression of *HHIP-AS1* rescued the reduced proliferation and viability (Fig. S3f), highlighting that *HHIP-AS1* is not only aberrantly overexpressed in SHH-driven tumors, but also functionally relevant in these malignancies. Conversely, transfection of *HHIP-AS1* siRNAs into non SHH MB cells (HD-MB03 and CHLA-01) with low expression of *HHIP-AS1* (Fig. S3g) did not result in reduced proliferation and viability (Fig. S3h), ruling out potential off-target effects and confirming that the functional relevance of *HHIP-AS1* is restricted to SHH-driven tumors. Remarkably, *HHIP* expression was not affected on mRNA or protein levels upon stable *HHIP-AS1* depletion (Fig. S4a–c). Therefore, we rule out a cis-regulatory effect of *HHIP-AS1* on *HHIP* in our MB and ATRT models.

### Identification and functional validation of *HHIP-AS1* downstream targets reveals that *HHIP-AS1* binds to the mRNA of *DYNC1I2*

**Fig. 3:**
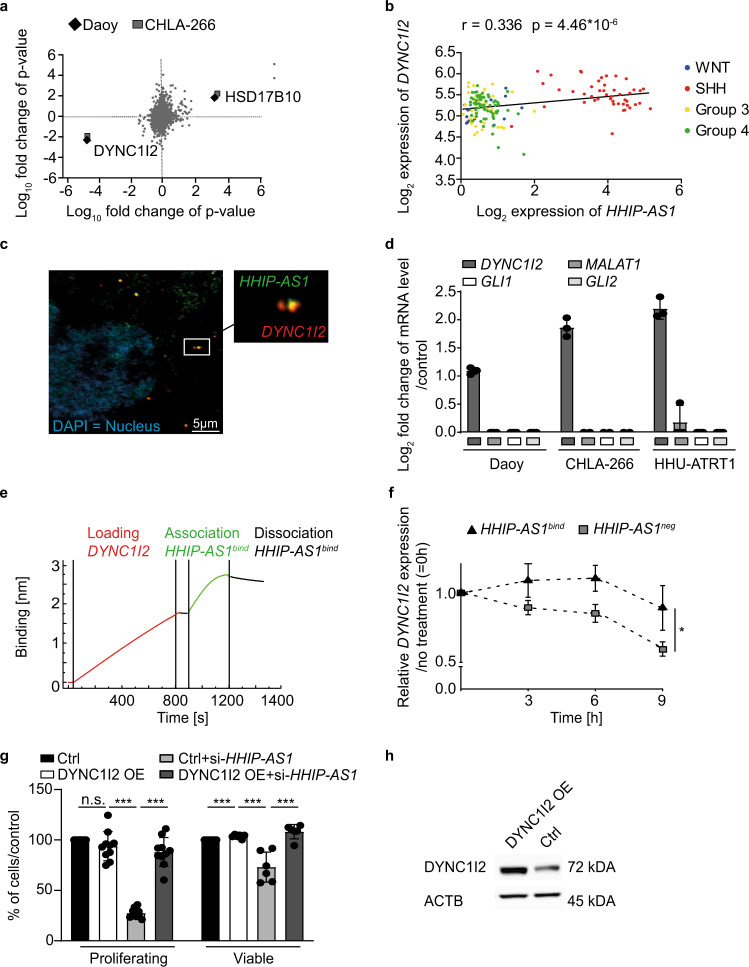
Identification and functional validation of *HHIP-AS1* downstream targets reveals that *HHIP-AS1* binds to mRNA of *DYNC1I2*. **a** Scatter plot indicates the correlation analysis of RNA sequencing (*x*-axis) and protein mass spectrometry (y-axis) data in two different cell models (Daoy and CHLA-266) upon *HHIP-AS1* knockdown (using sh-*HHIP-AS1#1* and sh-*HHIP-AS1#2*) *versus* control cells (sh-*scr*, *n* = 3 independent samples per condition and cell model). **b** Scatter plot displaying expression correlation of *DYNC1I2* and *HHIP-AS1* sequencing data comparing FPKM expression values in 167 MB patient samples. Samples are color coded for MB subgroups. Statistics were done by Pearson correlation. **c** Representative image of co-localization of *HHIP-AS1* and *DYNC1I2* mRNA in Daoy obtained through two-color fluorescence in situ hybridization (FISH). White frame indicates the location of the zoom out picture at the right side. Green: *HHIP-AS1* lncRNA, red: *DYNC1I2* mRNA, blue: DAPI, Nucleus. Scale bar: 5 µm. This experiment was repeated twice with similar results. **d** Enrichment of *DYNC1I2* mRNA upon *HHIP-AS1* raPOOL pulldown in Daoy and CHLA-266 cell lines and HHU-ATRT1 primary cells. Bar graphs are presented as the mean ± SD of three independent experiments. **e** Bio-Layer interferometry was used for detecting direct interaction between *DYNC1I2* mRNA and *HHIP-AS1*. **f** *DYNC1I2* mRNA stability upon transfection of a control (*HHIP-AS1*^*neg*^) or the *HHIP-AS1* interacting sequence (*HHIP-AS1*^*bind*^). Calculation was done in comparison to the mRNA level at time point “0 h” in each condition. Data are presented as the mean ± SEM of five independent experiments; Student´s two-sided *t*-test; *p < 0.05. **g** Bar graph indicating the proliferation rate or viability of Daoy cells in control condition (Ctrl), upon overexpression of DYNC1I2 (DYNC1I2 OE) and upon transient *HHIP-AS1*-knockdown (si-*HHIP-AS1*) in DYNC1I2 overexpression (DYNC1I2 OE + si-*HHIP-AS1*). Data are represented as the mean ± SD of at least six independent experiments normalized to the control condition. Student’s two-sided *t*-test; ***p < 0.001; n.s. not significant. **h** The immunoblot shows a representative blot of DYNC1I2 protein expression in control (Ctrl) or DYNC1I2-overexpressing (DYNC1I2 OE) cells. ACTB immunoblotting was used as loading control. This experiment was done twice with similar result. Source data and exact *p*-values are provided as a “Source Data file”.

In order to decipher the molecular mechanism controlled by *HHIP-AS1*, we investigated the impact of *HHIP-AS1* knockdown on both transcriptome and proteome in two independent cell models (Daoy and CHLA-266). Interestingly, our integrative proteogenomic analysis identified two candidates that were highly and consistently perturbed upon *HHIP-AS1* knockdown in both models, namely hydroxysteroid 17-beta dehydrogenase 10 (HSD17B10) and DYNC1I2 (Fig. 3a, RNA sequencing data: NCBI GenBank #GSE140741 and proteomic data: ProteomeXchange PRIDE database #PXD016550). We decided to focus on *DYNC1I2* based on its co-expression with *HHIP-AS1* in primary MB samples (*r* = 0.336; *p* < 0.001, Fig. 3b), as well as its high expression level specifically in SHH MB subgroup (Fig. S5a) and its known functions in neurodevelopment and cell cycle progression^42^. *HSD17B10* did not show a significant correlation with *HHIP-AS1* in primary MB samples (Fig. S5b). We validated by qRT-PCR and immunoblots that *HHIP-AS1* depletion reduced the mRNA and protein expression of DYNC1I2 in Daoy, CHLA-266 and HHU-ATRT1 cells (Fig. S5c and d). To determine the underlying molecular mechanism, we investigated a potential interaction between *HHIP-AS1* and *DYNC1I2* mRNA. First, we uncovered that both RNAs are co-localized in cells using RNA fluorescence in situ hybridization (Fig. 3c and S5e) and this co-localization can be enhanced or disrupted via SHH activation or inhibition, respectively (Fig. S5f). Second, we confirmed a direct interaction in each of our three cell models using an RNA-RNA-centric-pulldown probe set directed against *HHIP-AS1* RNA, which led to a specific enrichment for *DYNC1I2* mRNA compared to negative controls (Fig. 3d). Finally, we computationally derived the sequence of *HHIP-AS1* that is predicted to pair with *DYNC1I2* mRNA (Supplementary Table S1), identifying a 24 nucleotides long region (named *HHIP-AS1*^*bind*^) able to bind the 5'UTR of *DYNC1I2*. Furthermore, using bio-layer interferometry, we found that *HHIP-AS1*^*bind*^ could physically interact with *DYNC1I2* mRNA (Fig. 3e). In contrast, a negative control sequence of *HHIP-AS1* (named *HHIP-AS1*^*neg*^), carrying the same GC content and number of nucleotides as *HHIP-AS1*^*bind*^, but devoid of any in silico pairing potential to *DYNC1I2* mRNA, did not show any binding activity (Fig. S5g). Strikingly, we found that in vitro transfection of Daoy or HHU-ATRT1 with *HHIP-AS1*^*bind*^ resulted in extended half-life of *DYNC1I2* mRNA (Fig. 3f and S5h), compared to a control condition where *HHIP-AS1*^*neg*^ was used. All these findings confirm the existence of a functional and direct physical interaction between these two RNAs in vivo and in vitro, which ultimately regulates *DYNC1I2* expression levels. In order to elucidate whether the functional interaction between *HHIP-AS1* and *DYNC1I2* mediates the pro-proliferating phenotype controlled by *HHIP-AS1*, we evaluated the functional impact of *DYNC1I2* depletion in our cell models. Interestingly, transient *DYNC1I2* knockdown resulted in reduced proliferation and viability to a similar degree as observed upon *HHIP-AS1* depletion (Fig. S6a and b). More importantly, when we overexpressed DYNC1I2 using CRISPR-Cas9-based activation in *HHIP-AS1*-transiently depleted cells, both proliferation and viability were restored (Fig. 3g and h), indicating that *HHIP-AS1* exerts its pro-proliferative effects by controlling *DYNC1I2* abundance in tumor cells.

### The interaction between *HHIP-AS1* and *DYNC1I2* promotes mitosis

**Fig. 4:**
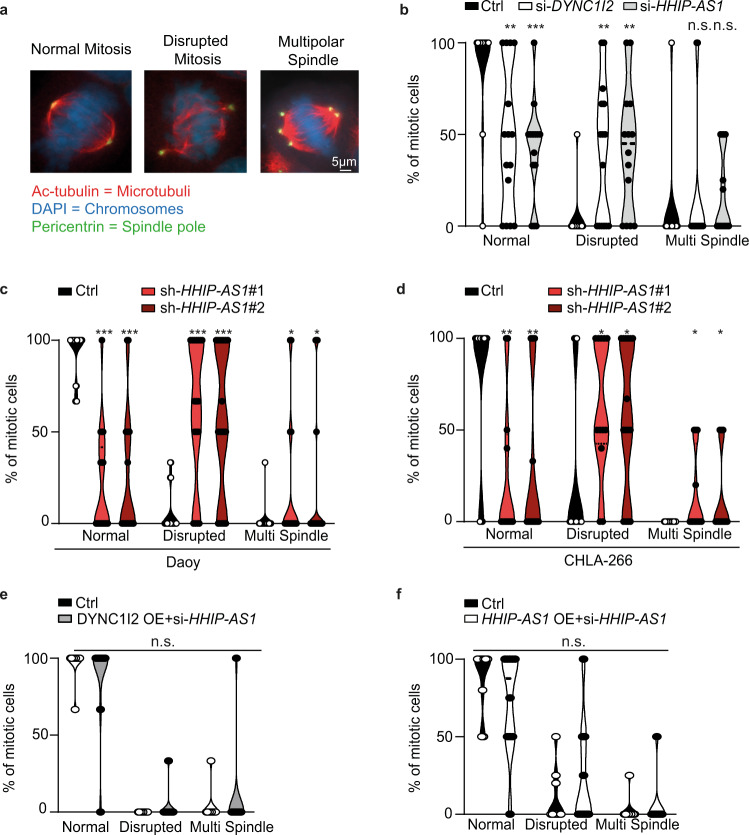
The interaction between *HHIP-AS1* and *DYNC1I2* promotes mitosis. **a** Representative images of immunofluorescence analysis show spindle assembly in mitotic cells by immunostaining for acetylated tubulin (Ac-tubulin, red) and pericentrin (green). Chromosomes are visualized with DAPI (blue). White scale bar: 5 μm. **b**–**d** Bar graphs display the percentage of dividing cells displaying normal, disrupted or multipolar spindle mitosis under control (Ctrl = si-*negative*-POOL or sh-*scr*) condition and *DYNC1I2*- or *HHIP-AS1*-knockdown using siRNAs for transient knockdown in Daoy (**b**) or two independent shRNAs (sh-*HHIP-AS1*#1 and sh-*HHIP-AS1*#2) for stable *HHIP-AS1* knockdown in Daoy (**c**) and in CHLA-266 (**d**). **e** Bar graphs showing the percentage of dividing cells displaying normal, disrupted or multipolar spindle mitosis under control (Ctrl) condition or upon rescue of DYNC1I2 expression by endogenous gene transcriptional activation through CRISPR-Cas9 technology in the context of transient *HHIP-AS1* knockdown (DYNC1I2 OE + si-*HHIP-AS1*) in Daoy cells. **f** Bar graphs showing the percentage of dividing cells displaying normal, disrupted or multipolar spindle mitosis under control (Ctrl) condition or upon rescue of *HHIP-AS1* expression by endogenous gene transcriptional activation through CRISPR-Cas9 technology in the context of transient *HHIP-AS1* knockdown (*HHIP-AS1* OE + si-*HHIP-AS1*) in Daoy cells. All bar graph values are representative of *n*  at least  ten independent experiments (with a total *n* > 50 counted mitotic cells) and data are shown as mean ± SEM. Student’s two-sided *t*-test; ***p < 0.001; **p < 0.01; *p < 0.05; n.s. not significant. Source data and exact *p*-values are provided as a “Source Data file”.

Cytoplasmic dynein-complex 1 has been implicated in various phenotypes including cargo transport on cytoplasmic microtubules and it was recently reported that *DYNC1I2* loss disrupts mitotic spindle organization in zebrafish neural progenitor cells^42^. Thus, we hypothesized that *HHIP-AS1* loss may cause a similar effect in human tumor cells by reducing DYNC1I2 availability. Indeed, we found that transient knockdown of *HHIP-AS1* or *DYNC1I2* via siRNAs transfection significantly and consistently altered mitotic spindle organization in cells compared to siRNA control-transfected cells (Fig. 4a and b; Fig. S6c and d), leading to more DNA damage in the mitotic cells as well (Fig. S6e). Moreover, using two different shRNAs against *HHIP-AS1*, we could confirm the alteration in mitotic spindle organization in Daoy and CHLA-266 (Fig. 4c and d) upon stable *HHIP-AS1* depletion. More importantly, induced overexpression of *DYNC1I2* as well as of *HHIP-AS1* with CRISPR-Cas9-based activation, rescued the mitotic spindle organization (Fig. 4e and f) in the context of transient *HHIP-AS1* knockdown, supporting that *HHIP-AS1* promotes proliferation through mitotic spindle stabilization by controlling DYNC1I2 abundance.

### *HHIP-AS1* blocks endogenous hsa-miR-425-5p function to maintain DYNC1I2 level

**Fig. 5:**
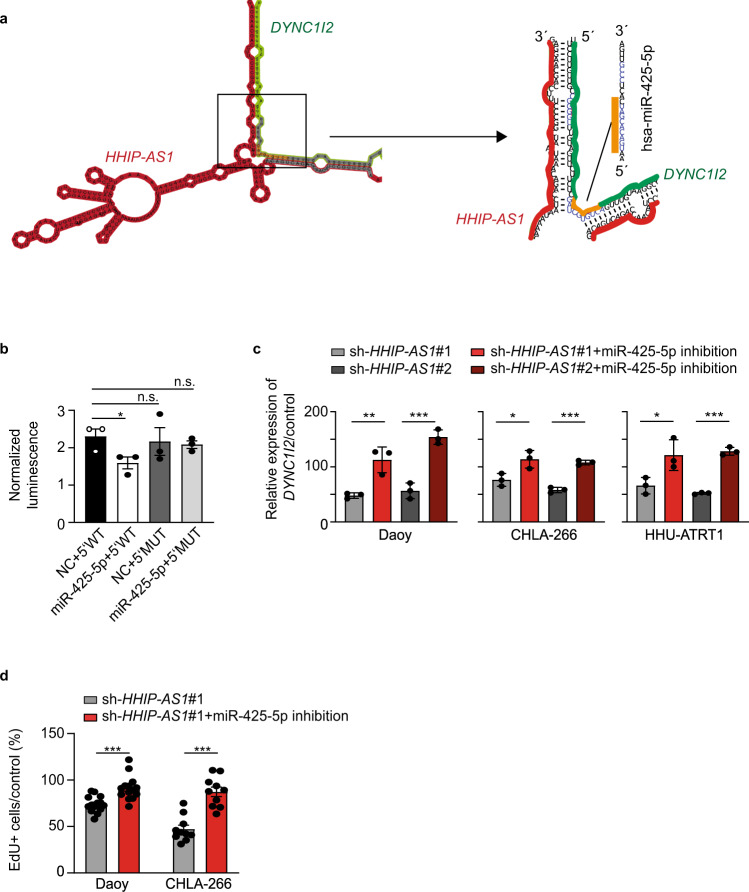
*HHIP-AS1* blocks endogenous hsa-miR-425-5p function to maintain *DYNC1I2* levels. **a** Schematic view of the complex structure generated by the predicted base-pairing interaction between *HHIP-AS1* (red) and the 5'UTR of *DYNC1I2* mRNA (green). The sequence of hsa-miR-425-5p is also shown and the inferred binding site of this miRNA on *DYNC1I2* mRNA 5'UTR is depicted in orange. The scheme highlights how this miRNA-mRNA interaction is predicted to be blocked by *HHIP-AS1* association to *DYNC1I2* mRNA. Black frame indicates position for zoom out image on the right side. **b** The wild type 5'UTR of *DYNC1I2* mRNA (5'WT) or a mutated version (5'MUT) were cloned in front of a luciferase coding sequence and co-transfected in combination with hsa-miR-425-5p mimics (mir-425) or negative controls (NC) into Daoy cells. Bar graphs indicate the measured relative luciferase activity for each combination ± SEM in three independent experiments. Student’s one-sided *t*-test; *p < 0.05. n.s. not significant. **c** *DYNC1I2* expression level was measured via qRT-PCR in Daoy, CHLA-266 and HHU-ATRT1 cells upon stable *HHIP-AS1* knockdown using two independent stable shRNAs (sh-*HHIP-AS1*#1 and sh-*HHIP-AS1*#2), in combination with or without transient inhibition of hsa-miR-425-5p. Bar graphs are presented as the mean ± SD of three independent experiments. **d** Proliferation rate of Daoy and CHLA-266 with stable knockdown of *HHIP-AS1* (sh-*HHIP-AS1*#1) measured by EdU incorporation in combination with or without transient inhibition of hsa-miR-425-5p. Bar graphs are presented as the mean ± SEM of at least ten independent experiments and corresponding controls were set to 100%. Student’s two-sided *t*-test; ***p < 0.001; **p < 0.01; *p < 0.05; n.s. not significant. Source data and exact *p*-values are provided as a “Source Data file”.

To further mechanistically elucidate how *HHIP-AS1* stabilizes *DYNC1I2* mRNA, we evaluated the genetic sequence of RNA-RNA interaction. Our analysis revealed six potential miRNA binding sites (Supplementary Table S2) within the predicted interaction region between *HHIP-AS1* and *DYNC1I2* mRNA (Fig. 5a), suggesting that *HHIP-AS1* binding to *DYNC1I2* mRNA may interfere with miRNA-dependent regulation of *DYNC1I2* expression. Therefore, we correlated the expression of these miRNA candidates with *DYNC1I2* using RNA sequencing data of 167 primary MB samples. Out of these six miRNAs, four were not detected in MB patient samples, while two miRNAs showed expression in MB patient samples, namely hsa-miR-425-5p (see binding site in Fig. 5a) and hsa-miR-1915. Notably, hsa-miR-425-5p expression level showed no differences across molecular subgroups of MB patients and cell models (Fig. S7a and b), but demonstrated anti-correlation with *DYNC1I2* expression in patient samples (*r* = −0.312; *p* < 0.001, Fig. S7c) compared to hsa-miR-1915 (Fig. S7d). After dividing the dataset into SHH and non SHH MB, the anti-correlation was only maintained in non SHH MB samples (*r* = −0.303; *p* < 0.001, Fig. S7e). We therefore tested whether a functional relationship existed between hsa-miR-425-5p and *DYNC1I2*. First, we cloned the 5´UTR of *DYNC1I2* in front of a luciferase reporter and found that hsa-miR-425-5p functionally binds this sequence causing a reduction of luciferase expression (Fig. 5b), hence confirming our previous in silico prediction (Fig. 5a). Importantly, this effect was abrogated upon mutation of the miRNA binding sequence on *DYNC1I2* 5´UTR (Fig. 5b). Second, we demonstrated that hsa-miR-425-5p inhibition significantly increased *DYNC1I2* mRNA levels upon stable *HHIP-AS1* knockdown in three SHH-driven cell models, while transfection of a negative control miRNA inhibitor did not restore *DYNC1I2* expression (Fig. 5c). This effect could be also observed after in vitro transfection of stable *sh-HHIP-AS1* Daoy with only *HHIP-AS1*^*bind*^ sequence resulting in higher expression of *DYNC1I2* mRNA independent of hsa-miR-425-5p inhibition (Fig. S8a), compared to a control condition where *HHIP-AS1*^*neg*^ was used. Interestingly, the corresponding regulatory element of *DYNC1I2*, where hsa-miRNA-425-5p is binding, is not evolutionary conserved in mice (Fig. S9). Third, inhibition of hsa-miR-425-5p rescued the decreased proliferation phenotype in Daoy and CHLA-266 obtained with *HHIP-AS1* knockdown (Fig. 5d). Finally, blockage of hsa-miR-425-3p showed no effect on *DYNC1I2* expression level (Fig. S8b–d).

Thus, our data unravel a regulatory network requiring *HHIP-AS1* to bind to *DYNC1I2* mRNA, to prevent *DYNC1I2* depletion mediated by binding of hsa-miRNA-425-5p to its 5'UTR. Together, our correlative data analysis approach in primary MB samples combined with in silico predictions and mechanistic validation, uniformly point to a previously poorly explored regulatory function of lncRNAs which consists in binding to a specific mRNA and thereby blocking endogenous miRNA binding sites.

### Loss of *HHIP-AS1* extends survival in SHH-driven brain tumors in vivo

**Fig. 6:**
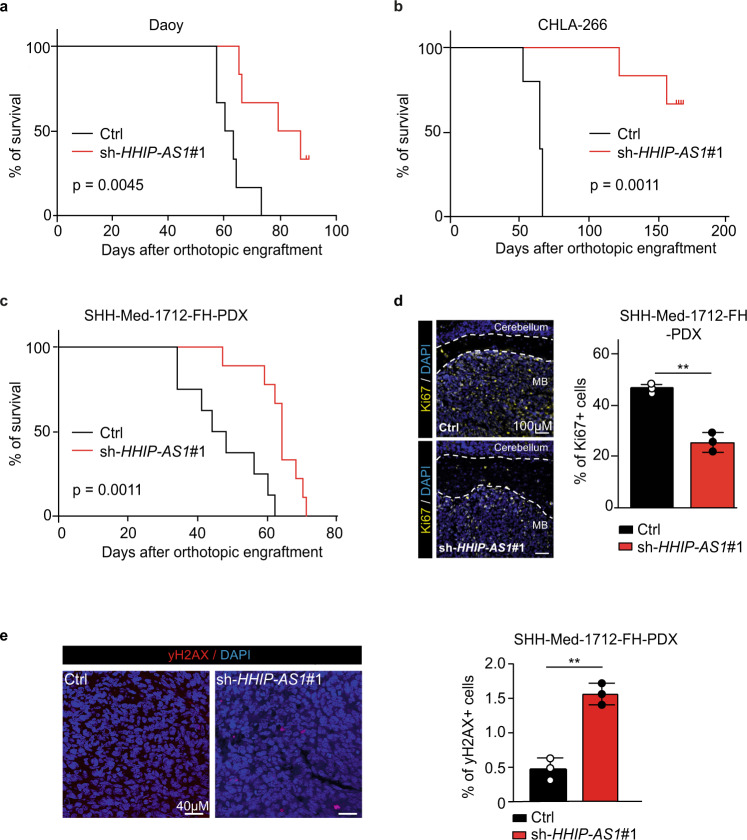
Loss of *HHIP-AS1* extends survival in SHH-driven brain tumor models in vivo. **a**,**b** Kaplan–Meier estimates of nude mice orthotopically engrafted with stably *HHIP-AS1*-silenced (sh-*HHIP-AS1*#1) Daoy (**a**, *n* = 13 mice) and CHLA-266 (**b**, *n* = 13 mice) cells compared to corresponding controls (sh-*scr*), respectively. **c** Survival curves of nude mice orthotopically engrafted with transiently *HHIP-AS1*-silenced (sh-*HHIP-AS1*#1) SHH-Med-1712-FH cells (*n* = 17mice). For panels **a**, **b**, and **c**, the median survivals were compared using Mantel–Cox test. **d,e** Three controls and three *HHIP-AS1*-depleted SHH-Med-1712-FH tumors were immunostained for Ki67 (yellow, **d**, white scale bar: 100 µm), γH2AX (red, **e**, white scale bar: 40 µm) and the percentage of positive stained tumor cells is plotted in the bar graphs as mean ± SD. Nuclei are stained with DAPI (blue). Both, control (ctrl = sh-*scr*) and sh-*HHIP-AS1*#1 tumor tissue, are shown in the two representative images. Student’s two-sided *t*-test, **p < 0.01. Source data and exact *p*-values are provided as a “Source Data file”.

We next evaluated whether targeting *HHIP-AS1* affected tumor growth in vivo. To this end, we initially utilized two well-established orthotopic brain tumor models with aberrant SHH signaling activation. Remarkably, stable knockdown of *HHIP-AS1* in both orthotopically engrafted Daoy and CHLA-266 cells significantly extended the survival of recipient mice compared to corresponding isogenic controls (Daoy, *p* = 0.0045; CHLA-266, *p* = 0.0011; Fig. 6a and b). Repeated luminescence measurement indicated that the loss of *HHIP-AS1* affected tumor formation in vivo consistently (Fig. S10a–c). We next aimed at validating our finding using a well-characterized patient-derived xenograft model of SHH MB. We transduced cells from the SHH-Med-1712-FH PDX model in vitro with sh-*HHIP-AS1* or control shRNA prior to transplantation into the cerebella of recipient mice. Knockdown of *HHIP-AS1* and corresponding *DYCN1I2* reduction was confirmed in this model via qRT-PCR (Fig. S10d). Interestingly, silencing of *HHIP-AS1* in injected PDX cells delayed the appearance of signs of morbidity in animals, whose mean survival was significantly extended compared to control mice (*p* = 0.0011; Fig. 6c). Lastly, *HHIP-AS1* depletion markedly reduced cell proliferation index in Med-1712-FH PDX tumors compared to control tumors, when these samples were tested for Ki67-immunoreactivity (Fig. 6d). Accordingly, we observed more differentiated cells in tumor tissue compared to control (Fig. S11a). In line with our in vitro data, we detected no change in caspase activity (cleaved caspase) in Med-1712-FH PDX tumors (Fig. S10e and S11b). However, we observed more DNA damage in tumor tissue compared to control (Fig. 6e) and in NSC (Fig. S9f) after *HHIP-AS1* depletion. Overall, these results provide compelling evidence that *HHIP-AS1* promotes tumorigenicity of SHH-driven human tumors in vivo.

## Discussion

Until now the complexity of the human SHH signaling pathway and regulatory feedback loops is not fully understood. Specifically, the precise knowledge of the molecular mechanisms underlying SHH pathway pro-oncogenic activity in human cells is urgently required. To this aim, the widespread use of genetic animal models of SHH-driven malignancies has been successful at identifying and describing a large number of protein-coding genes and protein post-translational modifications implicated in regulation and downstream functions of SHH signaling in tumors^43–46^. Nevertheless, despite accounting for ~70% of the transcribed genome in humans^47^, mechanistic insights into the role of lncRNAs in SHH-driven cancer biology have been gained only for a very few species. A reason for this poor consideration relies on the general low level of inter-species conservation of lncRNAs, which has inevitably hindered their discovery and functional characterization in the commonly used genetic rodent models. In our study, we employed a large comparative transcriptome analysis approach across several thousands of human normal and cancerous tissues, and identified *HHIP-AS1* as a lncRNA that is specifically upregulated in SHH-driven tumors and functionally required for mediating the pro-proliferative effects of oncogenic SHH signaling. Interestingly, we found that *HHIP-AS1* constitutes a downstream transcriptional target of SHH signaling, providing an explanation to its restricted and elevated expression in SHH-driven entities. Specifically, we uncovered that *HHIP-AS1* transcription is initiated from a bidirectional promoter shared with the known SHH-pathway regulator *HHIP*. However, unlike *HHIP* or other key protein-coding gene of the SHH pathway, *HHIP-AS1* is poorly conserved across vertebrates, showing high sequence similarity between humans and other primates, but substantially no conservation between humans and rodents. Therefore, our findings pinpoint the importance of species-specific evaluation of oncogenic signaling pathways, and emphasize how some key regulatory networks or pro-oncogenic programs may not be faithfully recapitulated by rodent tumor models, despite their generally well-accepted value to study human pathogenic processes. Furthermore, by using a combined proteomic and RNA sequencing approach, we uncovered that *HHIP-AS1* acts in trans by regulating the expression of *DYNC1I2*, which encodes for an intermediate chain of the cytoplasmic dynein-complex 1, and not *in cis* by regulating *HHIP*^48^. We, among others, could already demonstrate the power of integrated proteogenomic approaches, showing that proteomic combined with transcriptomic profiles provides a profound insight into active oncogenic gene expression regulation in pediatric brain tumors and other cancers^16,25^. The involvement of the cytoplasmic dynein-complex 1 provides an intriguing aspect of SHH pathway regulation and function. Indeed, dynein motor complexes have been long implicated in the activity of SHH signaling. However, this association was only restricted to cytoplasmic dynein-complex 2, due to its requirement at the level of primary cilia in mediating ciliary retrograde transport^49^. By characterizing the functional interaction between *HHIP-AS1* and *DYNC1I2* in cancer cells and in NSC, we unraveled the existence of an additonal layer whereby SHH signaling sustains cell growth and progression. SHH signaling is already known to directly promote cell cycle progression by primarily activating the expression of *MYCN* and *Cyclin D* genes, which drive cells toward the G1/S transition^50^. By sustaining the transcription of *HHIP-AS1*, the SHH signaling guarantees effective spindle assembly and chromosome segregation through maintenance of appropriate levels of DYNC1I2. Interestingly, we could observe in cells with *HHIP-AS1* depletion that the dysregulation of mitosis led to more DNA damage in mitotic cells. Although the DNA damage response and the mitotic cell division pathways were thought to be distinct and unrelated, it was shown in several studies that mitotic cells can experience DNA damage either endogenously due to unrepaired premitotic damage or exogenously from cancer therapies amongst other causes^51–53^. As we demonstrate that SHH exerts a control on cell division during the time of mitosis in cancer cells and in NSC, therapeutic limitations may exist. Targeting the SHH signaling pathway seems to provide a highly innovative therapeutic option for a broad variety of cancers. Nevertheless, as SHH pathway inhibitors seem to be safe in adults, this still remains to be shown in children treated for MB and taking into consideration our findings in NSCs. While the introduction of targeted therapies represents an exciting era in personalized medicine, compounds that target developmental signaling pathways (including SHH signaling) should be carefully evaluated for specific toxicities and their potential benefits.

Depleting cells of *HHIP-AS1* or blocking its interaction with *DYNC1I2* mRNA exposes the latter to hsa-miRNA-425-5p*-*mediated degradation, thereby compromising mitotic fidelity and consequently blocking cell progression in vitro and in vivo. As the power of miRNA in regulation of mRNA has been explored elaborately, we are beginning to recognize also the complex interactions of miRNAs and lncRNAs and ensuing RNA regulatory networks. Our work provides a previously unmatched molecular understanding of lncRNA function by actively competing with a miRNA binding site on a target mRNA. We show that the lncRNA *HHIP-AS1* constitutes a so far unexplored target gene of SHH signaling, which enables the pro-mitotic effects of this pathway by blocking hsa-miR-425-5p-dependent inhibition of DYNC1I2 expression. This mechanism is of fundamental interest as it describes not only a major mediator of cell progression triggered by SHH activation but also as provides a previously unknown epigenetic regulation of SHH pathway in humans. However, to comprehensively understand the role of the regulatory *HHIP-AS1/DYNC1I2/miR425* axis and its potent pro-mitotic effects, further experiments are required to elucidate the relevance in SHH-driven neurogenesis and tumorigenesis.

## Methods

### Animal models

For the orthotopic brain tumor models we used 8 week old female *NMRI*-*Foxn1nu*/*nu* that were purchased from Janvier Labs, Le Genest-Saint-Isle, France. We followed both European and national regulations for animal housing, care and experimentation (Directive 86/609). The use of animals was approved by the reporting ethical committee and the ministry under the agreement #03130.20. Ethics committee: CCEA-IC, Instances: Higher Education of the Ministry for Education and Research (France).

### Cell culture

The cell line Daoy was obtained from American Type Culture Collection (ATCC, Manassas, VA) and was grown in Dulbecco’s Modified Eagle’s medium (DMEM) + GlutaMAX-I high glucose medium (Thermo Fisher Scientific, Waltham, MA, #31966021) supplemented with 10% fetal bovine serum (FBS) (Merck, Darmstadt, Germany #F9665) and 1% penicillin/streptomycin (P/S) (Merck, #P4333). Cell line CHLA-266 was purchased from CCcells (Childhood Cancer Repository) and was grown in 1x Iscove’s Modified Dulbecco’s medium (IMDM; Thermo Fisher Scientific, #12440053) supplemented with 20% FBS, 4 mM L-glutamine (Thermo Fisher Scientific, #25030032) and 1% insulin transferrin selenium (Thermo Fisher Scientific, #41400045). HHU-ATRT1 cells were generated at the University Hospital Düsseldorf (ethical permission #2018-102) and were grown in Neurobasal-A medium (Thermo Fisher Scientific, #10888022) complemented with 2 mM L-glutamine, 1% P/S, 0,0075% bovine serum albumin (BSA), B-27 supplement 1x (Thermo Fisher Scientific, #12587001), heparin (4 µg/ml, StemCell, #07980), recombinant epidermal growth factor (EGF, 10 ng/ml, Thermo Fisher Scientific, #RP-10914) and basic fibroblast growth factor (FGF, 10 ng/ml, Biomol, #HZ-1285). The cell line HD-MB03 was a generous gift of Dr. Till Milde, KiTZ, Hopp Children´s Cancer Center Heidelberg, and was grown in RPMI1640 (Thermo Fisher Scientific, #22400089) supplemented with 10% FBS and 1% MEM non-essential amino acids (Thermo Fisher Scientific, #11140050). Cell lines CHLA-01 and CHLA-04 were purchased from ATCC (CHLA-01, #ATCC CRL-3021 and CHLA-04, #ATCC CRL-3036) and grown in DMEM/F12 (Thermo Fisher Scientific, #11330-032) complemented with 2% B27, 20 ng/mL EGF and FGF. Neuronal stem cells (NSCs, H9 hESC-Derived) were purchased from Thermo Fischer Scientific (#GIBCO N7800100) and cultivated according to manufactures’ protocol. The cell line RH30 was a generous gift of Prof Simone Fulda, Head of Christian-Albrechts-University Kiel, Germany, and was grown in RPMI1640 (Thermo Fisher Scientific, #22400089) complemented with 10% FBS, 1% P/S, 1% Sodium Pyruvate (100 mM, Thermo Fisher Scientific, #11360-070). UI226 cell line was a generous gift of Prof Anthony Oro and Dr. Francois Kuonen, Department of Dermatology, Stanford University, USA, and was grown in KO-DMEM F12 cell culture media (Thermo Fisher Scientific, #A10509-01) and complemented with 1x supplement (Thermo Fisher Scientific, # A10509-01), 200nM L-glutamine and 20 ng/ml of FGF and EGF. All cell lines were incubated at 37 °C and 5% CO_2_. For SHH activation or inhibition, parental cells were treated with 50 nM smoothened agonist (Merck, #566661) or 1 µM cyclopamine (MedChemExpress, Sollentuna, Sweden, #HY-17024), respectively, for 24 h. SHH MB PDX lines, namely ICN-MB12^54^ and Med-1712-FH^55^ were grown in vitro in Neurobasal-A medium supplemented with 2 mM L-glutamine, 1% P/S, 0.45% D-glucose (Merck, #G7528), 0.4% BSA (Merck, #A9165), B27 and N-acetyl-L-cysteine (Merck, #A9576). Orthotopic engraftment was performed as previously described^54^. PDX cells were cultured in vitro exclusively for the time required for transducing them with lentiviruses. Daoy cells were co-infected with sh-*HHIP-AS1* and MSCV-IRES-Luciferase lentiviruses prior orthotopic injection. Tumor growth was followed by Xenogen Ivis Spectrum imaging system (Perkin-Elmer). Patient-derived MB cells were freshly isolated from tissue samples obtained by surgical resection at the Department of Neurosurgery, University Hospital Düsseldorf with informed consent by the patients and approval by the institutional review board (study number: 2018-102). Cells were cultivated for 3 days before transient siRNA knockdown of *HHIP-AS1* was performed.

### Transient siRNA and miRNA transfection

Parental Daoy, CHLA-266, HHU-ATRT1, HD-MB03, CHLA-01, CHLA-04, RH30, NSC and patient-derived MB cells were transfected with 10 nmol siPOOLs (*HHIP-AS1, DYNC1I2*, see “Oligonucleotides” section in Table S3) using Lipofectamine RNAiMAX reagent (Thermo Fisher Scientific, #13778150) and harvested for RNA isolation after 48 and/or 72 h after transfection or were used for further analyses including EdU incorporation, colony formation assays, CTG and immunoblotting. For miRNA inhibition experiments, shRNA models of each cell line (included scramble as control, sh-*HHIP-AS1*#1 and sh-*HHIP-AS1*#2) were seeded into six-well plates. After reaching about 80% confluency, cells were transfected using Lipofectamine RNAiMAX either with the miRNA inhibitor for miR-425-5p, miR-425-3p or the negative miRNA inhibitor control (see “Oligonucleotides” in Table S3).

### shRNA cloning and lentiviral transduction

shRNAs of indicated sequences (see “Oligonucleotides” in Table S3) were cloned into pLK0.1-TRC-Puro (Addgene, Watertown, MA, #10878). Pseudotyped lentivirus was generated as previously described^31^, and HEK293T cells were transfected with a packaging plasmid, envelope plasmid, and the generated shRNA vector plasmid using polyethylenimine (Merck, #408727). Virus was collected 48 h and 72 h after transfection, 0.4 μm filtered, and used directly for transduction with polybrene (Merck, #TR-1003-G). Successfully infected cells were selected in puromycin (0.8–1.5 μg/ml; Merck, #P8833) containing medium.

### Overexpression

For the overexpression of *DYNC1I2*, *GLI* and *HHIP-AS1* in Daoy, we used a transcriptional activation by an engineered CRISPR-Cas9 complex referred to as “SAM”. Cloning of SAM sgRNA was performed as previously described^56^; for sequence details, see “gDYNC OE”, “gGLI OE” and “g*HHIP-AS1* OE” in “Oligonucleotides” section in Table S3. After stable transduction of the sgRNAs, we performed a transient knockdown of *HHIP-AS1* as described in section “Transient siRNA and miRNA transfection”.

### RNA extraction, cDNA synthesis and qRT-PCR

Total RNA was extracted using TRIzol (Thermo Fisher Scientific, #15596018) according to the manufacturer’s instructions or by using the MaxWell system (Promega, #AS1340). Isolated RNA was quantified by spectrophotometry and 500 ng of RNA was retrotranscribed to total cDNA using random hexamer and oligo-dT primer mix (Promega, Madison, WI, #C110A), dNTP mix (Promega, #U1511), M-MLV-RT 5X buffer (Promega, #M3681), M-MLV RT, RNase (H–), point mutant (Promega, #M368C) and RNasin-Plus ribonuclease inhibitor (Promega, #N2511). Resulting cDNA was analyzed by TaqMan-based qRT-PCR containing FAM-labeled probes. All reactions were performed in triplicates using a “CFX384 Touch Real-Time PCR Detection System” (Bio-Rad, Hercules, CA). Relative expression levels of candidate genes were acquired by normalization against the housekeeper genes *GUSB*, *HPRT1* and *PPIA*.

### MiRNA extraction, cDNA synthesis and qRT-PCR

MicroRNAs (miRNA) were extracted using the “Maxwell RSC miRNA Kit” (Promega, #AS1470) according to the manufacturer’s instructions. Afterwards the “Applied Biosystems™ TaqMan™ Advanced miRNA cDNA Synthesis Kit” (Thermo Fisher Scientific, #A28007) was used according to the manufacturer’s instructions. For qRT-PCR analyses singleplex miRNA Assays (Thermo Fisher Scientific) for hsa-miR-425-5p (SM-10204) and hsa-miR191 (SM-20786) as a control were used.

### Primers, siRNAs, raPOOLs and shRNAs

TaqMan-based primers were purchased from IDTdna: *DYNC1I2* (Hs.PT.58.3736795); *GLI1* (Hs.PT.58.26486279), *GLI2* (Hs.PT.58.45642781); *GUSB* (Hs.PT.58 v.27737538); *HHIP* (Hs.PT.58.40948312); *HHIP-AS1* (Hs.PT.58.613732); *HPRT1* (Hs.PT.58 v.45621572); *MALAT1* (Hs.PT.58.26451167.g); *PPIA* (Hs.PT.39a.22214851); *ppia* (Mm.PT.39a.2.gs)*, b2m* (Mm.PT.39a.22214835)*, gusb* (Mm.PT.39a.22214848)*, hhip* (Mm.PT.58.29299649) and *gli1* (Mm.PT.58.11933824). siRNA pools, consisting of 30 different siRNAs, were purchased from siTOOLs Biotech (Martinsried, Germany): *HHIP-AS1* (#646576- 10 nmol); *DYNC1I2* (#1781- 10 nmol); raPOOLs, consisting of 30 biotinylated RNA probes, were also from siTOOLs Biotech: *HHIP-AS1* (#raPOOL646576 –5 nmol).

### Immunoblots

Immunoblots experiments were performed using the following primary antibodies: anti-DYNC1I2 (Atlas Antibodies, Bromma, Sweden, #HPA040619, 1:1000), mouse anti-β-Actin (C4) (Santa Cruz Biotechnology, Heidelberg, Germany, #sc-47778, 1:1000) or mouse anti-β-Actin (Cell Signaling Technology, Danvers, MA, #3700, 1:1000), mouse anti-HHIP (Abnova Germany; #H00064399-M01; 1:1000); mouse anti-GLI2 (Santa Cruz Biotechnology, Heidelberg, Germany, #sc-271786, 1:500); rabbit anti-GLI1 (Cell Signaling Technology, Danvers, MA, #V812,1:1000).

### Luciferase reporter assay

Promoter insert (Chromosome 4: 144,645,400-144,647,801; hg19 coordinates) was subcloned into pGL4.22 [luc2CP/Puro] Vector (Promega, #DQ188841) in two orientations. The forward sequence (“HHIP-fw”) was flipped around for the “HHIP-rv” sequence. The correctness of insert orientations was confirmed by sequencing. The reporter activity was measured by using “Dual-Luciferase Reporter Assay System” (Promega, #E1910) according to the manufacturer’s instructions. The 3'- and 5'-Luciferase reporter gene assays were performed as described previously^57^ except for transfecting cells with 50 nM of pre-miR miRNA precursor hsa-miR-425-5p (Thermo Fisher Scientific, #17100) and control cells with 50 nM of the pre-miR miRNA precursor negative control #1 (Thermo Fisher Scientific, #AM17110).

### RNA fluorescence in situ hybridization (FISH)

FISH was carried out based on the Stellaris RNA FISH protocol according to the manufactory. Daoy and CHLA-266 cells were fixed in 4% formaldehyde (Merck, #100496.8350) at room temperature for 10 min. Cells were permeabilized in 70% ethanol at 4 °C for 1 h followed by hybridization with FITC-labeled *HHIP-AS* and ATTO655-labeled *DYN1I2* probes in hybridization buffer (10% formamide, 10% dextran sulfate, 2x saline sodium citrate) at 37 °C overnight. Nuclei were stained with 4', 6-diamidino-2-phenylindole (DAPI). Images were acquired using a wide field fluorescence microscope Axio Observer.Z1 (Carl Zeiss Microscopy, Jena, Germany) with an ApoTome 2 (Zeiss) attachment.

### Bio-Layer interferometry

Bio-Layer interferometry (BLI) has been a promising technique to study the RNA-RNA binding interaction^58^. We used the “Fortebio Blitz System” (Fortebio, Fremont, CA) to study the interaction between the proposed RNA sequences along with a negative control sequence. We used 1x RNA binding buffer (RBB) containing 10 mM Tris-HCl pH 8.0, 125 mM NaCl, 125 mM KCl & 25 mM MgCl_2_ to perform all binding studies. We used a modified protocol from^58^ to study the binding interaction in our system. Basically, SSA biosensor tips were hydrated in RBB for at least 10 min. Next, 200 nM of biotinylated *DYNC1I2* sequence (ordered from IDTdna, see section “In silico analysis of RNA binding”) was loaded for 15 min after performing an initial baseline of the unloaded sensors for 30 s in RBB. Subsequently, the loaded sensors were incubated in RBB for 1 min. The loaded sensors were incubated with 200 nM of *HHIP-AS1* pos/neg sequence (ordered from IDTdna, see section “In silico analysis of RNA binding”) for 5 min in the association phase, and dissociation of the bound sequences was studied for 5 min again in RBB. All the RNase free buffer components were purchased from “Thermo Fisher Scientific”. The biotinylated and non-biotinylated RNA sequences were ordered from IDTdna. The Super-Streptavidin (SSA) coated biosensors were purchased from Fortebio.

### Immunofluorescence for cell proliferation, DNA damage and mitotic spindle evaluation

For immunofluorescence experiments, parental Daoy, CHLA-266, HHU-ATRT1, RH30 and NSC cells were seeded on 8-well glass slides (Nunc™ Lab-Tek™ II Chamber Slide™ System, Thermo Fisher Scientific, #154534). The next day, cells were transfected using siPOOLs as described above for transient knockdown. Stable generated shRNA models for each cell line (included *scr* as control, sh-*HHIP-AS1*#1 and sh-*HHIP-AS1*#2) were also seeded on 8-well glass slides. SHH MB PDX cells of ICN-MB12 were seeded on 8-well glass slides (Millicell EZ slide, Merck, #PEZGS0816) pre-coated with poly-D-lysine (Merck, #A-003-E) and Matrigel (Dutscher, Brumath, France #354230). Lentiviral transduction with shRNA constructs was performed 2 h after seeding. Cells were maintained in culture for 10 days, passing them when confluence was reached. A pulse of 10 μM BrdU (BD Biosciences, #550891) was provided before fixation with 4% PFA for 20 min. For orthotopic SHH MB tumors of Med-1712-FH PDX line, whole mouse brains bearing the tumor were fixed for 10 h in 4% PFA. The tissue was then embedded in paraffin and sagittal sections (3 μm) were obtained using microtome (Leica, Wetzlar, Germany). Proliferation analysis in Daoy, CHLA-266, HHU-ATRT1 and patient-derived MB cells was performed 72 h after transient, siRNA-mediated knockdown of *HHIP-AS1* using the “Click-iT EdU Alexa-Fluor 488 Imaging Kit” (Thermo Fisher Scientific, #C10337) according to the manufacturer’s instructions. PDX cells in vitro and orthotopic PDX tissue sections were instead stained with 1:400 rabbit anti-Ki67 (Merck Millipore, Darmstadt, Germany, #AB9260), 1:1000 rabbit anti-NeuN (Abcam, #ab177487), 1:100 Rabbit anti-Cleaved Caspase 3 (D175, Cell Signaling, #9661S), 1:100 mouse anti-phospho-γH2AX (JBW301,Merck Millipore, Darmstadt, Germany, #05-636-I) for in vivo tissue slides or 1:400 rabbit anti-phospho-histone γH2A.X (Cell Signaling, Frankfurt, Germany #9718) for in vitro staining and 1:500 anti-BrdU (Bio-Rad AbD Serotec, Oxford, UK, #OBT0030G) primary antibodies. For DNA damage in vitro studies, neuronal stem cells or Daoy were seeded on 8-well glass slides (Nunc™ Lab-Tek™ II Chamber Slide™ System, Thermo Fisher Scientific, #154534) fixed in paraformaldehyde, rinsed in phosphate-buffered saline, and incubated with γH2AX antibodies and then Alexa 568-conjugated goat anti-rabbit IgG as secondary antibody. Slides mounted with Slow-Fade antifade reagent were imaged on an Zeiss inverted Apotome microscope with a ×63 oil immersion objective (Zeiss). Before counting foci, digital images were processed with ImageJ (LOCI, University of Wisconsin) to adjust brightness and contrast. Cells were evaluated as “positive” for γH2AX foci if they displayed >10 discrete dots of brightness. For tumor sections, only distinct and bright cells for γH2AX were counted as positive cells. Mitotic spindle staining was achieved by staining cells with 1:500 rabbit anti-pericentrin (Abcam, Cambridge, UK, #AB 4448**)** and 1:500 mouse anti-acetylated tubulin (Merck, #T6793) primary antibodies, detecting the centrosomes and the microtubules of the spindle, respectively.

In all cases, secondary antibodies were species-specific: chicken anti-rabbit IgG (H + L) cross-adsorbed secondary antibody, Alexa-Fluor-488 labeled (Thermo Fisher Scientific, #A-21441) and goat anti-mouse IgG2b cross-adsorbed secondary antibody, Alexa-Fluor-594 labeled (Thermo Fisher Scientific, #A-21145).

### Clonogenic, caspase 3/7 activity and cell viability assays

For the analysis of self-renewal capacity, clonogenicity was analyzed. Daoy, CHLA-266 and HHU-ATRT1 were seeded on 10 cm dishes at the appropriate density and cultured for 1–3 weeks. For transient knockdown approach, cells were transfected with siPOOL against *HHIP-AS1* as described above after 24 h of seeding. Stable shRNA models of each cell line (included control, sh-*HHIP-AS1*#1 and sh-*HHIP-AS1*#2) were also seeded in 10 cm dishes at the appropriate density and cultured for 1–3 weeks. The cells were washed with PBS, fixed in 10% formaldehyde for 30 min at RT and stained in 1% crystal violet for 1 h at RT. Next, cells were washed in ddH_2_O and the number of colonies was quantified. Caspase 3/7 activity was measured using Caspase-Glo 3/7 Assay System (Promega, Walldorf, Germany, #G8091) and cell viability assessment was determined by metabolic assay, using CellTiter-Glo Luminescent Cell Viability Assay (Promega, Walldorf, Germany, #G7570) according to the manufacturer’s instructions.

### In silico analysis of RNA binding

The potential binding between *HHIP-AS1* and *DYNC1I2* mRNA was analyzed in silico using IntaRNA^59,60^ and http://rtools.cbrc.jp/ bioinformatics web servers. We detected a 24 nt long sequence (CCCTTGCCTACAACCAGACTGACA) in *HHIP-AS1*, which binds to the 5´UTR region of *DYNC1I2*. This sequence we used as a “positive binding” probe for mRNA stability assay and we designed another sequence with a similar G/C content from *HHIP-AS1* that was predicted not to bind *DYNC1I2*, as a negative control (TTCAGCCTCCAAGGGGGCTTTTAA). Secondary structures of two RNAs forming dimers were predicted with RNACOFOLD^61^, which takes into account intra- as well as intermolecular base pairs of both sequences. Secondary structures of single sequences were predicted with RNAFOLD. All calculations were performed at *T* = 37 °C and all used programs were from the “ViennaRNA package” v. 2.4.6^62^.

### mRNA stability assay

Cells were pretreated either in the presence or absence of positive binding sequence (*HHIP-AS1*^*bind*^; see “in silico analysis of RNA binding”) or “not binding” sequence (*HHIP-AS1*^*neg*^) before the addition of actinomycin D (Hycultek; 10 μg/ml final concentration), a potent inhibitor of mRNA synthesis. Afterwards, total mRNA was extracted at 0–9 h and *DYNC1I2* abundance was measured by qRT-PCR.

### RNA sequencing and conservation analysis

For RNA sequencing analysis, reads generated from Daoy scr (control), Daoy sh-*HHIP-AS1*#1, Daoy sh-*HHIP-AS1*#2, CHLA-266 scr (control), CHLA-266 sh-*HHIP-AS1*#1and CHLA-266 sh-*HHIP-AS1*#2 were filtered, normalized and aligned to the human genome hg38 using STAR (v2.4.1d), unaligned reads were further aligned using BOWTIE2 (v2.2.5) and combined reads were quantified using the partek expectation-maximization algorithm against ENSEMBL release 84. For conservation analyses across species, the genomic loci and surrounding genomic regions for the species were analyzed using ECR browser (https://ecrbrowser.dcode.org/) and Ensembl (https://www.ensembl.org/index.html). Annotated species-specific exonic and intronic regions are highlighted in yellow or orange, respectively. Used RefSeq datasets were: zebrafish [danRer7], chicken [galGal3], rat [rn4], mouse [mm10], rhesus macaque [rheMac2], chimpanzee [panTro3] and human [hg19].

### Proteomic analyses

#### Sample preparation

Proteins were extracted from frozen cell pellets as previously described^63^. Briefly, cells were homogenized in urea buffer with a “TissueLyser” (Qiagen, Hilden, Germany) and subsequent sonication. After centrifugation for 15 min at 14,000 g and 4 °C, supernatants were collected. Protein concentration was determined via pierce 660 nm protein assay (Thermo Fisher Scientific) and 10 µg protein per sample were desalted through electrophoretic migration at 50 V for 10 min on a 4–12% bis-tris polyacrylamide gel (Thermo Fisher Scientific, #NP0322BOX). After silver staining, protein bands were cut out, reduced, alkylated and digested with trypsin before peptide extraction via sonication. Peptides were dissolved and diluted with 0.1% TFA (v/v).

#### LC-MS analysis

For mass spectrometric analysis, 15 µl peptide solution per sample was analyzed on a nano-high-performance liquid chromatography electrospray ionization mass spectrometer. The analytical system was composed of a “RSLCnano U3000 HPLC” coupled to a “Orbitrap Elite mass spectrometer via a nano-electrospray ion source” (Thermo Fisher Scientific). Injected peptides were concentrated and desalted at a flow rate of 6 µl/min on a trapping column (Acclaim PepMao C18, 2 cm × 100 µm × 3 µm particle size, 100 Å pore size, Thermo Fisher Scientific) with 0.1% TFA (v/v) for 10 min. Subsequently, peptides were separated at a constant flowrate of 300 nl/min over a 120 min gradient on an analytical column (Acclaim PepMap RSLC C18, 25 cm × 75 µm × 2 µm particle size, 100 Å pore size, Thermo Fisher Scientific) at 60 °C. Separation was achieved through a gradient from 4 to 40% solvent B (solvent A: 0.1% (v/v) formic acid in water, solvent B: 0.1% (v/v) formic acid, 84% (v/v) acetonitrile in water). Afterwards, peptides were ionized at a voltage of 1.400 V and introduced into the mass spectrometer operated in positive mode. Mass spectrometry scans were recorded in profile mode in a range from 350–1700 m/z at a resolution of 60,000 while tandem mass spectra were recorded in the ion trap at normal scan rate. Tandem mass spectra were recorded with a data dependent Top 20 method and 35% normalized collision energy. Dynamic exclusion was activated with a repeat count of 1 for 45 s and only charge states 2+ and 3+ were analyzed.

#### Computational mass spectrometric data analysis

Proteome discoverer (version 1.4.1.14, Thermo Fisher Scientific) was applied for peptide/protein identification with mascot (version 2.4, Matrix Science) as search engine employing the UniProt database (human; including isoforms; date 2016-11-01). A false discovery rate of 1% (*p* ≤ 0.01) on peptide level was set as the identification threshold. Proteins were quantified with Progenesis QI for Proteomics (Version 2.0, Nonlinear Dynamics, Waters Corporation).

### Statistics

Unless otherwise indicated in the figure legends, error bars represent mean ± SD or SEM of at least three independent experiments for each genotype or sample, and the used statistical test is indicated in each figure legend. Graphs were generated by using GraphPad PRISM ® Version 9 Graphpad Software.

### Reporting summary

Further information on research design is available in the Nature Research Reporting Summary linked to this article.

## Supplementary information

Supplementary Information

Description of Additional Supplementary Files

Supplementary Data 1

Reporting Summary

## Data Availability

Previously published gene expression and methylation data used in Fig. 1a–e, 3b, S1b–f, S5a, b, S7a, c–e are available under ‘R2: Genomics Analysis and Visualization Platform (http://r2.amc.nl)’. RNA sequence data that support the findings of this study have been deposited in GenBank with the #GSE140741 accession code and proteomic data have been deposited in ProteomeXchange PRIDE database with the #PXD016550 accession code. The authors declare that the data supporting the findings of this study are available within the paper and its supplementary information files. Proteome discoverer (version 1.4.1.14, Thermo Fisher Scientific) was applied for peptide/protein identification with mascot (version 2.4, Matrix Science) as search engine employing the UniProt database (human; including isoforms; date 2016-11-01). The raw data behind data points in figures and that support the findings of this study are available in the linked. Source data provided with this paper.

## Source data

Source Data
