# Supplementary material for: The HHIP-AS1 lncRNA promotes tumorigenicity through stabilization of dynein complex 1 in human SHH-driven tumors
Source: Nat Commun. 2022 Jul 13;13:4061. doi: 10.1038/s41467-022-31574-z (PMC9279496; doi:10.1038/s41467-022-31574-z)
Supplement: Supplementary file 1 — Supplementary Information [file 41467_2022_31574_MOESM1_ESM.pdf]

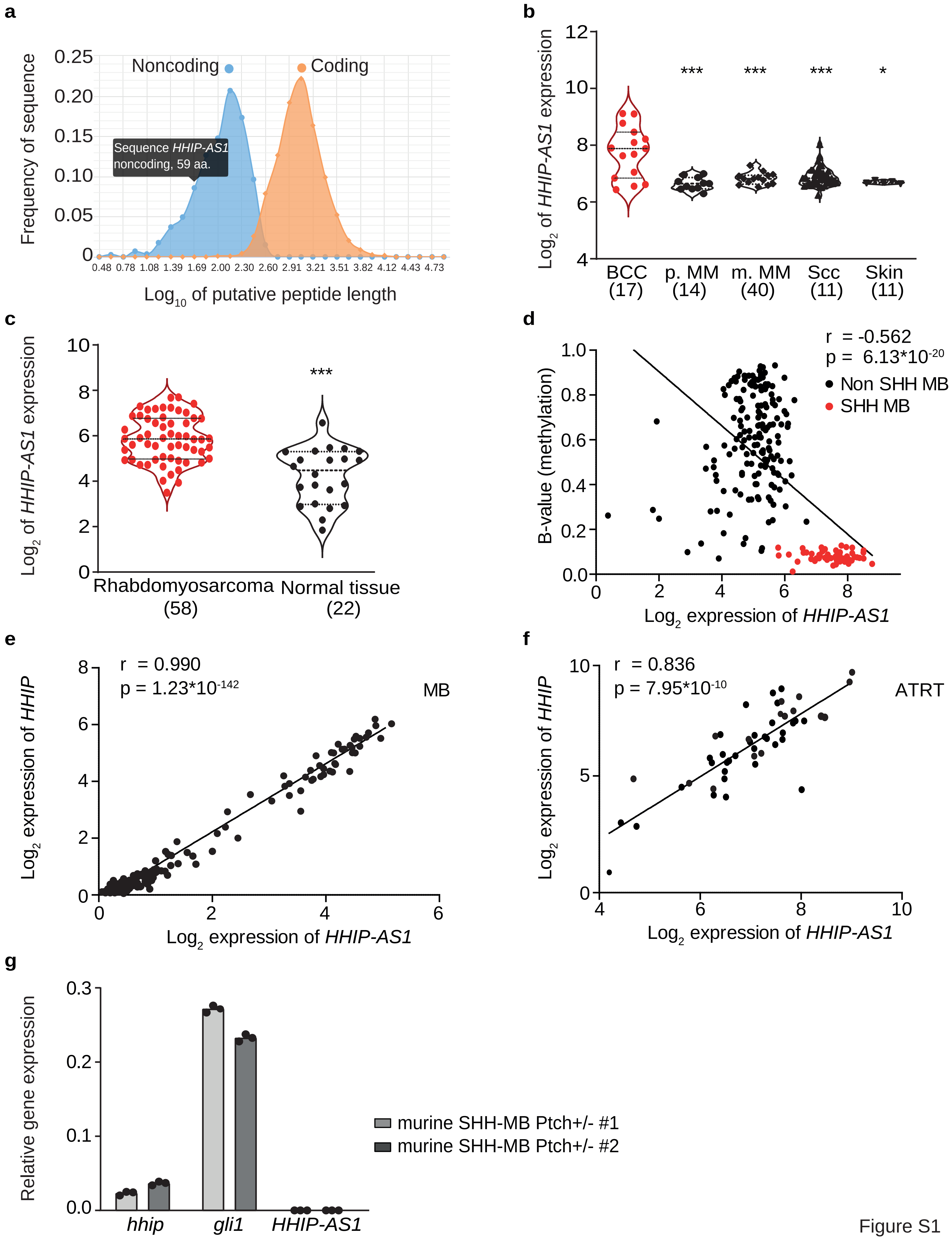
a
Noncoding
Coding
Frequency of sequence
0.25
0.20
0.15
0.10
0.05
0
Sequence HHIP-AS1
noncoding, 59 aa.
0.48 0.78 1.08 1.39 1.69 2.00 2.30 2.60 2.91 3.21 3.51 3.82 4.12 4.43 4.73
Log10 of putative peptide length
b
Log2 of HHIP-AS1 expression
12
10
8
6
4
***
***
***
*
BCC (17)
p. MM (14)
m. MM (40)
Scc (11)
Skin (11)
c
Log2 of HHIP-AS1 expression
10
8
6
4
2
0
***
Rhabdomyosarcoma (58)
Normal tissue (22)
d
r = -0.562
p = 6.13*10-20
Non SHH MB
SHH MB
B-value (methylation)
1.0
0.8
0.6
0.4
0.2
0.0
0
2
4
6
8
Log2 expression of HHIP-AS1
e
r = 0.990
p = 1.23*10-142
MB
Log2 expression of HHIP
8
6
4
2
0
0
2
4
6
Log2 expression of HHIP-AS1
f
r = 0.836
p = 7.95*10-10
ATRT
Log2 expression of HHIP
10
5
0
4
6
8
10
Log2 expression of HHIP-AS1
g
Relative gene expression
0.3
0.2
0.1
0.0
hhip
gli1
HHIP-AS1
murine SHH-MB Ptch+/- #1
murine SHH-MB Ptch+/- #2

Figure S1

Supplementary figure legends

Figure S1: Validation of the long non-coding RNA *HHIP-AS1* expression in SHH-dependent entities

(a) Validation of non-coding potential of *HHIP-AS1* using a protein-coding potential assessment tool based on a logistic regression model. The selected features include the quality of the ORF (open frame region) and Fickett Score [1]. The Fickett Score is used to evaluate each base's unequal content frequency and asymmetrical distribution in the positions of codons in one sequence. (b) Violin plots show *HHIP-AS1* expression in basal cell carcinoma (BCC, red dots), primary melanoma (p.MM), metastatic melanoma (m.MM), squamous cell carcinomas (Scc) and normal skin. Data were analyzed from R2 database cohorts. Statistical analysis was performed using ANOVA two-way with posthoc Bonferoni Test; ***p < 0.001, *p < 0.05. (c) Violin plots show *HHIP-AS1* expression in rhabdomyosarcoma (red dots) compared to normal soft tissue (black dots). Statistical analysis was done by using Student's two-sided *t*-test; ***p < 0.001. (d) Scatter plot representing the degree of DNA methylation (B-value) of the potential *HHIP-AS1* promoter region in relation to *HHIP-AS1* expression levels indicated by an additional TSS probe (cg26339943) in SHH MB (red dots) and non SHH MB subgroups (black dots). Statistic was done by using Pearson correlation coefficient. (e+f) Scatter plots displaying the correlation between mean *HHIP* (230135_at) and *HHIP-AS1* (236632_at) expression in 167 MB patient samples (e) and in 49 ATRT samples (f). Statistics were done by using Pearson correlation coefficient. (g) Gene expression of mouse *hhip* and *gli1* and human *HHIP-AS1* in brain tissue from murine SHH MB *ptch1*+/- mice measured by qRT-PCR (gene expression was normalized to mice housekeeping genes *ppia*, *b2m* and *gusb*). Source data of are provided as a "Source Data file".

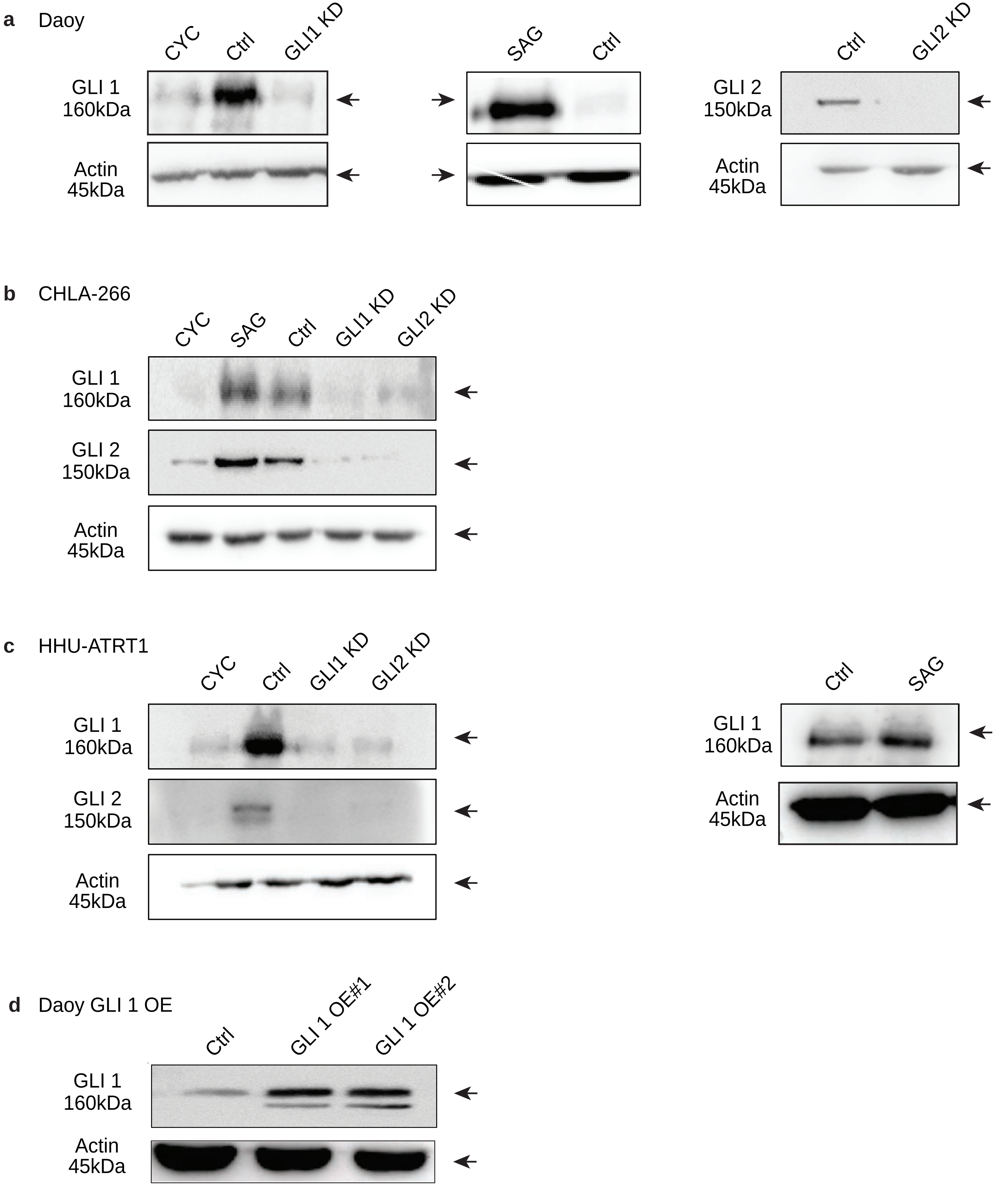

a
Daoy
CYC
Ctrl
GLI1 KD
GLI 1
160kDa
Actin
45kDa
SAG
Ctrl
Ctrl
GLI2 KD
GLI 2
150kDa
Actin
45kDa
b
CHLA-266
CYC
SAG
Ctrl
GLI1 KD
GLI2 KD
GLI 1
160kDa
GLI 2
150kDa
Actin
45kDa
c
HHU-ATRT1
CYC
Ctrl
GLI1 KD
GLI2 KD
GLI 1
160kDa
GLI 2
150kDa
Actin
45kDa
Ctrl
SAG
GLI 1
160kDa
Actin
45kDa
d
Daoy GLI 1 OE
Ctrl
GLI 1 OE#1
GLI 1 OE#2
GLI 1
160kDa
Actin
45kDa

Figure S2

Figure S2: Protein expression level of GLI 1 and GLI 2 after treatment

The images show representative immunoblots of GLI1 or GLI2 proteins in control (Ctrl), smoothened agonist (SAG) treated, cyclopamine (CYC) treated, GLI1 knockdown (KD) and GLI2 KD in Daoy (a), CHLA-266 (b) and HHU-ATRT cells (c) or GLI1 overexpression in Daoy (d). Actin immunoblotting was used as loading control. Source data of uncropped blots are provided as a "Source Data file". These experiments were performed three times with equal results.

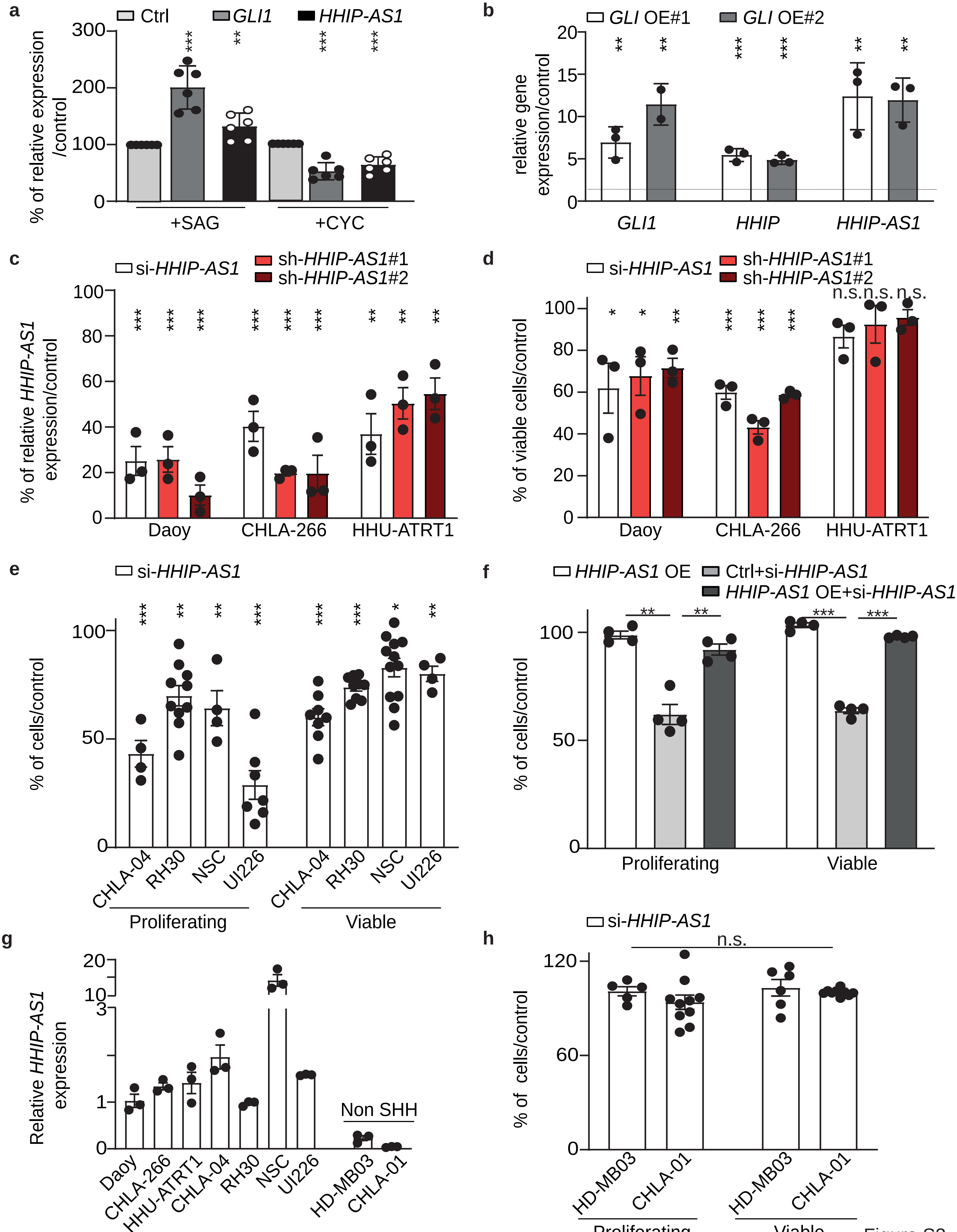
a
Ctrl
GLI1
HHIP-AS1
% of relative expression /control
+SAG
+CYC
b
GLI OE#1
GLI OE#2
relative gene expression/control
GLI1
HHIP
HHIP-AS1
c
si-HHIP-AS1
sh-HHIP-AS1#1
sh-HHIP-AS1#2
% of relative HHIP-AS1 expression/control
Daoy
CHLA-266
HHU-ATRT1
d
si-HHIP-AS1
sh-HHIP-AS1#1
sh-HHIP-AS1#2
n.s. n.s. n.s.
% of viable cells/control
Daoy
CHLA-266
HHU-ATRT1
e
si-HHIP-AS1
% of cells/control
CHLA-04
RH30
NSC
UI226
Proliferating
Viable
f
HHIP-AS1 OE
Ctrl+si-HHIP-AS1
HHIP-AS1 OE+si-HHIP-AS1
% of cells/control
Proliferating
Viable
g
Relative HHIP-AS1 expression
Daoy
CHLA-266
HHU-ATRT1
CHLA-04
RH30
NSC
UI226
Non SHH
HD-MB03
CHLA-01
h
si-HHIP-AS1
n.s.
% of cells/control
HD-MB03
CHLA-01
Proliferating
Viable

Figure S3

Figure S3: The expression of *HHIP-AS1* is SHH-dependent and is functionally relevant in human SHH-driven tumors

(a) The relative gene expression levels of *HHIP-AS1* and *GLI1* were tested in neuronal stem cells (NSC) upon pharmacological activation (SAG, smoothened agonist) or inhibition (CYC, cyclopamine). (b) Bar graphs showing relative gene expression of *GLI, HHIP* and *HHIP-AS1* in *GLI1* overexpression models in Daoy cells. (c) Transient (si-*HHIP-AS1*) or stable (sh-*HHIP-AS1#1* and sh-*HHIP-AS1#2*) knockdown efficacy of *HHIP-AS1* in Daoy, CHLA-266 and HHU-ATRT1 cells. Corresponding controls (either with si-*negative*-POOL or sh-*scr* transfected Daoy, CHLA-266 and HHU-ATRT1 cells) were set to 100% and expression levels of knockdowns were calculated accordingly. (d) Cell viability of Daoy, CHLA-266 and HHU-ATRT1 cells upon transient (si-*HHIP-AS1*) or stable knockdown (sh-*HHIP-AS1#1* and sh-*HHIP-AS1#2*) of *HHIP-AS1* normalized to controls. Corresponding controls (either si-*negative*-POOL or sh-*scr*) were set to 100% and cell viability of knockdowns was calculated accordingly. (e) Analysis of proliferation rate and cell viability upon transient knockdown of *HHIP-AS1* and si-*negative*-POOL control in CHLA-04 (ATRT), RH30 (rhabdomyosarcoma), neuronal stem cells (NSC) and UI226 (BCC) with high *HHIP-AS1* expression. Bar graphs illustrate EdU positive cells relative to the total counted cells (n = 300 cells were analyzed in each condition) and relative CellTiter-Glo signal to control cells. (f) Bar graph indicating the proliferation rate or viability of Daoy cells in control condition (Ctrl), upon overexpression of *HHIP-AS1* (*HHIP-AS1* OE) and upon transient *HHIP-AS1*-knockdown (si-*HHIP-AS1*) in *HHIP-AS1* overexpression (*HHIP-AS1* OE+si-*HHIP-AS1*). (g) Quantification of *HHIP-AS1* expression in the indicated tumor cell line models and NSC. (h) Analysis of proliferation rate and cell viability upon transient knockdown of *HHIP-AS1* and si-*negative*-POOL control HD-MB03 and CHLA-01 with low *HHIP-AS1* expression. Bar graphs illustrate EdU positive cells relative to the total counted cells ($n > 300$ cells were analyzed in each condition) and relative CellTiter-Glo signal to control cells. Results of panel a+b are presented as the mean ± SD and panel c-h are presented as the mean ± SEM of three or more independent experiments. Statistical analysis was done by using Student’s two-sided *t*-test; ***$p < 0.001$; **$p < 0.01$; *$p < 0.05$; n.s. = not significant. Source data and exact p-values are provided as a “Source Data file”.

a

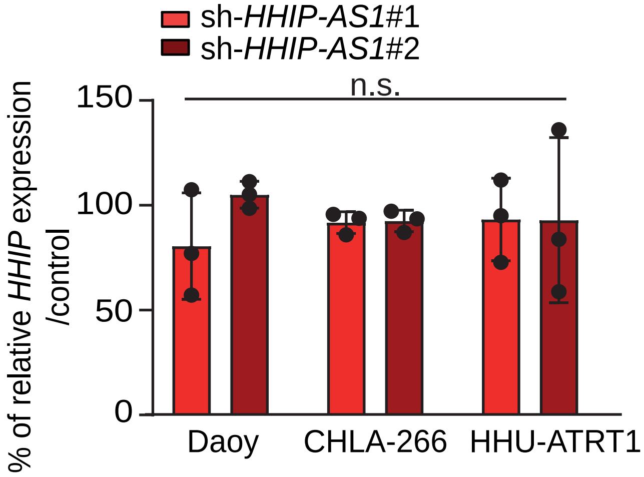

b

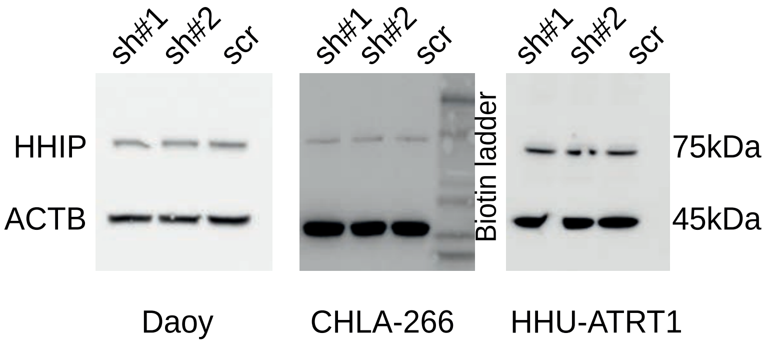

c

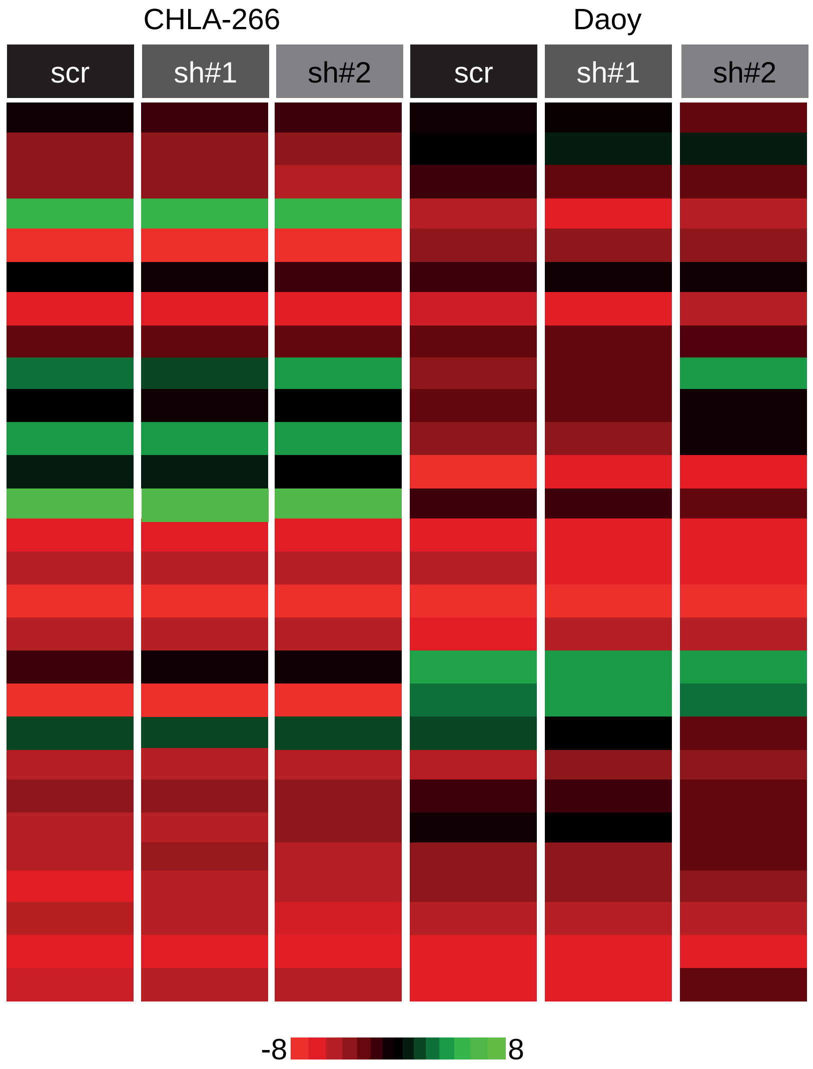

	sh#1		sh#2	
	**p value**	**FC**	**p value**	**FC**
HHIP	0.996	1.0	0.142	-2.6
GLI1	0.734	1.1	0.701	1.2
GLI2	0.842	-1.1	0.349	-1.4
GLI3	0.966	-1.0	0.531	1.1
PTCH1	0.750	1.0	0.993	-1.0
PTCH2	0.903	-1.0	0.737	-1.3
SMO	0.862	1.0	0.528	1.2
SUFU	0.711	1.1	0.708	1.1
BMP5	0.993	-1.0	0.431	17.8
WNT2B	0.806	-1.2	0.544	1.9
BMP2	0.162	-1.1	0.656	2.0
WNT5A	0.015	1.3	0.567	-1.2
BMP4	n/a	-1.1	0.356	-1.9
FBXW11	0.610	-1.1	0.905	1.0
CSNK1E	0.772	-1.1	0.380	-1.2
CSNK1A1	0.373	1.2	0.405	1.1
CSNK1G3	0.442	1.3	0.298	1.2
WNT9A	0.790	1.3	0.729	1.2
LRP2	0.799	1.2	0.996	1.0
WNT7B	0.598	-1.6	0.584	-3.8
BTRC	0.143	1.2	0.330	1.3
WNT3	0.302	-1.2	0.175	-1.4
WNT5B	0.364	1.2	0.704	-1.6
BMP7	0.692	1.3	0.057	1.5
PRKX	0.426	1.6	0.321	1.6
CSNK1G1	0.605	1.0	0.723	1.0
GSK3B	0.054	1.1	0.442	1.1
GAS1	0.274	1.1	0.429	4.4

Figure S4

Figure S4: Sonic hedgehog pathway genes expression is not affected upon *HHIP-AS1* depletion

(a) Gene expression level of *HHIP* in Daoy, CHLA-266 and HHU-ATRT1 cells measured by qRT-PCR after stable (sh-*HHIP-AS1#1* and sh-*HHIP-AS1#2*) knockdown of *HHIP-AS1* normalized to control. Corresponding control (sh-*scr* transfected Daoy, CHLA-266 and HHU-ATRT1 cells) was set to 100% and expression levels of knockdowns were calculated accordingly. Bar graphs are presented as the mean ± SD of three independent experiments. Statistical analysis was done by using Student's two-sided *t*-test; n.s. = not significant. (b) Protein expression level of HHIP in Daoy, CHLA-266 and HHU-ATRT1 cells measured by immunoblotting after stable knockdown of *HHIP-AS1* (sh-*HHIP-AS1#1* and sh-*HHIP-AS1#2*). A representative blot of HHIP protein expression in control (scr) or *HHIP-AS1* knockdown cells (sh#1 and sh#2) for each cell model is shown. ACTB immunoblotting was used as loading control. This experiment was repeated three times with similar results; see panel (a) for bar graph. Source data and exact p-values are provided as a "Source Data file". (c) Heatmap shows analysis of RNA sequencing and data in two different cell models (Daoy and CHLA-266) upon *HHIP-AS1* knockdown (using sh-*HHIP-AS1*#1 and sh-*HHIP-AS1*#2) *versus* control cells (sh-*scr*, n = 3 independent samples per condition and cell model). Selected SHH genes, based on KEGG pathway enrichment, are visualized as a relative color-coded scheme for each row according to the expression level. Statistical analysis was done by using Student's two-sided *t*-test.

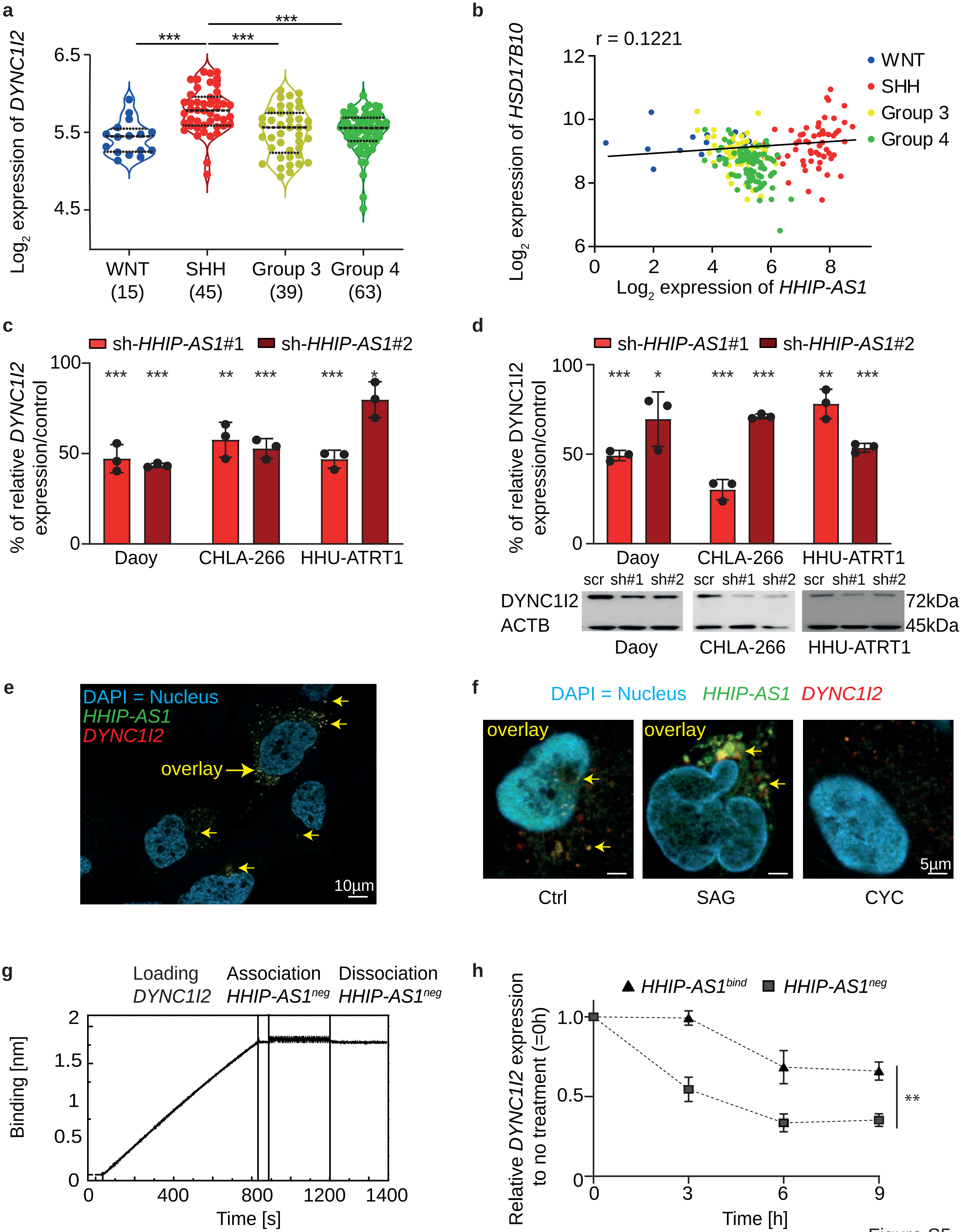
a
Log2 expression of DYNC1I2
WNT (15)
SHH (45)
Group 3 (39)
Group 4 (63)
b
r = 0.1221
Log2 expression of HSD17B10
Log2 expression of HHIP-AS1
WNT
SHH
Group 3
Group 4
c
sh-HHIP-AS1#1
sh-HHIP-AS1#2
% of relative DYNC1I2 expression/control
Daoy
CHLA-266
HHU-ATRT1
d
sh-HHIP-AS1#1
sh-HHIP-AS1#2
% of relative DYNC1I2 expression/control
Daoy
CHLA-266
HHU-ATRT1
scr sh#1 sh#2
DYNC1I2
ACTB
72kDa
45kDa
e
DAPI = Nucleus
HHIP-AS1
DYNC1I2
overlay
10µm
f
DAPI = Nucleus HHIP-AS1 DYNC1I2
overlay
5µm
Ctrl
SAG
CYC
g
Loading DYNC1I2
Association HHIP-AS1neg
Dissociation HHIP-AS1neg
Binding [nm]
Time [s]
h
HHIP-AS1bind
HHIP-AS1neg
Relative DYNC1I2 expression to no treatment (=0h)
Time [h]

Figure S5

Figure S5: ***DYNC1I2*** **comprises a target of** ***HHIP-AS1*** **and mediates its function**

(a) Violin plots represent the expression levels of *DYNC1I2* according to an integrative transcriptomic analysis of medulloblastoma (MB) patients (n = 162) divided into molecular subgroups. Statistical analysis was performed using one-way ANOVA with post-hoc Bonferroni; ***p < 0.001. (b) Scatter plot displaying expression correlation of *HSD17B10* and *HHIP-AS1* based on RNA sequencing data comparing fragments per kilobase million (FPKM) expression values in 167 patient samples in non SHH MB and SHH MB tumors. (c+d) Relative expression of *DYNC1I2* in cells upon stable *HHIP-AS1* knockdown with two independent shRNAs (sh-*HHIP-AS1*#1 and sh-*HHIP-AS1*#2) normalized to control (sh-*scr*). (c) Bar graph depicting *DYNC1I2* expression upon *HHIP-AS1* knockdown. (d) Bar graphs show relative DYNC1I2 protein expression level after stable *HHIP-AS1* knockdown (sh-*HHIP-AS1#1* and sh-*HHIP-AS1#2*) in indicated cell lines. Protein level was determined by densitometric analysis on three independent immunoblot experiments. The immunoblots on the lower panel show representative blots of DYNC1I2 in control (sh-*scr*) or *HHIP-AS1* (sh#1 and sh#2) knockdown cells. ACTB immunoblotting was used as loading control. (e+f) Representative image of co-localization of *HHIP-AS1* and *DYNC1I2* mRNA in a Daoy cell obtained through two-color fluorescence *in situ* hybridization (FISH). Yellow arrows indicate co-localization (overlay) of *HHIP-AS1* lncRNA and *DYNC1I2* mRNA under normal conditions (e +f, Ctrl) and after treatment with SHH pathway activator smoothened agonist (f, SAG) or inhibitor cyclopamine (f, CYC). Green: *HHIP-AS1* lncRNA, red: *DYNC1I2* mRNA, blue: DAPI, Nucleus. This experiment was done twice with similar results. (g) Bio-Layer interferometry was used for detecting direct interaction between *DYNC1I2* mRNA and *HHIP-AS1*. (h) *DYNC1I2* mRNA stability upon transfection of a control (*HHIP-AS1*^{neg}) or the *HHIP-AS1* interacting sequence (*HHIP-AS1*^{bind}). Calculation was done in comparison to mRNA level at time point "0 h" in each condition. Data in panel c+d are presented as the mean ± SD of three independent experiments; data in panel h are presented as the mean ± SEM of five independent experiments. Statistical analysis was performed using Student´s two-sided *t*-test, if not stated otherwise; ***p < 0.001; **p < 0.01; *p < 0.05. Source data and exact p-values are provided as a "Source Data file".

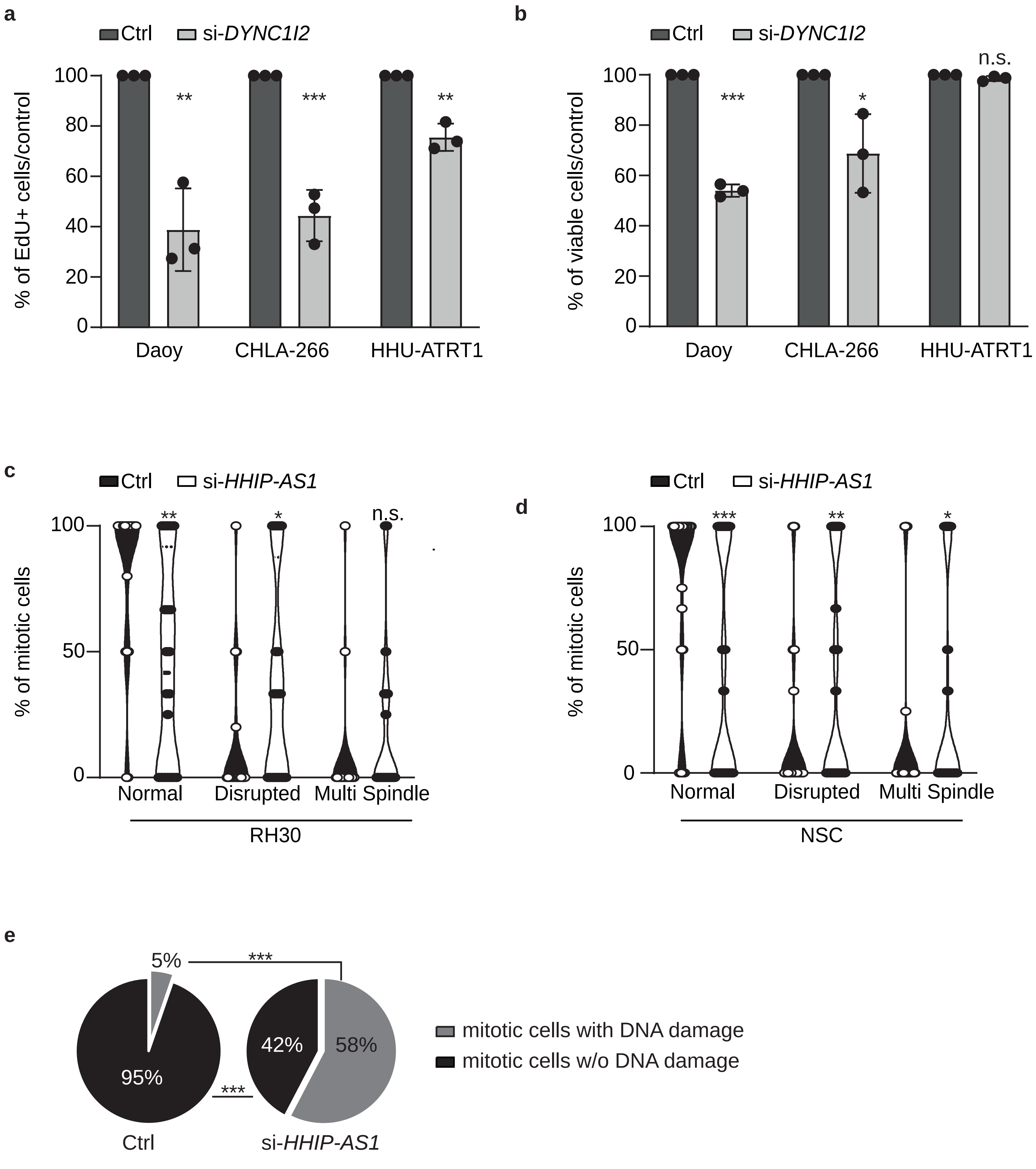
a
Ctrl
si-DYNC1I2
% of EdU+ cells/control
100
80
60
40
20
0
**
***
**
Daoy
CHLA-266
HHU-ATRT1
b
Ctrl
si-DYNC1I2
% of viable cells/control
100
80
60
40
20
0
***
*
n.s.
Daoy
CHLA-266
HHU-ATRT1
c
Ctrl
si-HHIP-AS1
% of mitotic cells
100
50
0
**
*
n.s.
Normal
Disrupted
Multi Spindle
RH30
d
Ctrl
si-HHIP-AS1
% of mitotic cells
100
50
0
***
**
*
Normal
Disrupted
Multi Spindle
NSC
e
5%
***
95%
42%
58%
***
Ctrl
si-HHIP-AS1
mitotic cells with DNA damage
mitotic cells w/o DNA damage

Figure S6

Figure S6: Transient knockdown of *DYNC1I2* corroborates phenotype of depletion of *HHIP-AS1* and verification of spindle formation in SHH-model after depletion of *HHIP-AS1*

(a) Bar graph indicates relative cell proliferation rate of Daoy, CHLA-266 and HHU-ATRT1 cells measured by EdU incorporation upon transient knockdown of *DYCNC1I2* (si-*DYNC1I2*) or control (Ctrl = si-*negative*-POOL). (b) Bar graph indicates relative cell viability of Daoy, CHLA-266 and HHU-ATRT1 cells measured by CellTiter-Glo upon transient knockdown of *DYNC1I2* (si-*DYNC1I2*) or control (Ctrl = si-*negative*-POOL). Bar graphs of panels a+b are presented as the mean ± SD of three independent experiments; statistical analysis was done by Student's *t*-test; ***p < 0.001; **p < 0.01; *p < 0.05; n.s.= not significant. (c+d) Bar graphs display the percentage of dividing cells displaying normal, disrupted or multipolar spindle mitosis under control (Ctrl = si-*negative*-POOL) condition and *HHIP-AS1* knockdown using siRNAs for transient knockdown in RH30 (c) and in neuronal stem cells (NSC) (d). (e) Pie charts display percentage of mitotic cells with or without DNA damage under control (Ctrl = si-*negative*-POOL) condition or *HHIP-AS1* knockdown in Daoy (n > 35 counted mitotic cells per condition). In panel c+d values are representative of n > 50 counted mitotic cells per condition and data are shown as mean ± SEM. Statistical analysis was done by Student's two-sided *t*-test; ***p < 0.001; **p < 0.01; *p < 0.05; n.s.= not significant. Source data and exact p-values are provided as a "Source Data file".

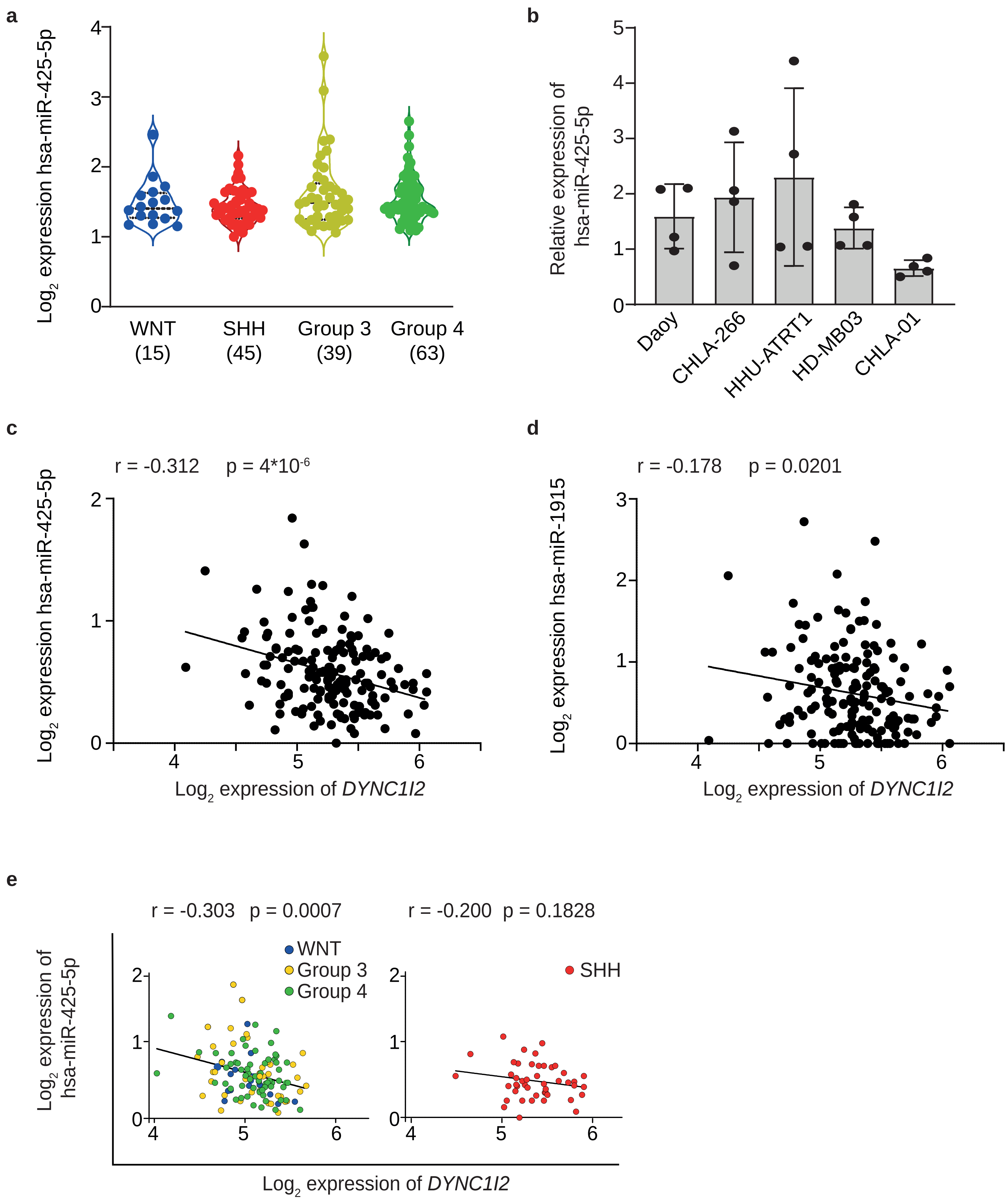

a
Log2 expression hsa-miR-425-5p
WNT (15)
SHH (45)
Group 3 (39)
Group 4 (63)
b
Relative expression of hsa-miR-425-5p
Daoy
CHLA-266
HHU-ATRT1
HD-MB03
CHLA-01
c
r = -0.312 p = 4*10-6
Log2 expression hsa-miR-425-5p
Log2 expression of DYNC1I2
d
r = -0.178 p = 0.0201
Log2 expression hsa-miR-1915
Log2 expression of DYNC1I2
e
r = -0.303 p = 0.0007
r = -0.200 p = 0.1828
WNT
Group 3
Group 4
SHH
Log2 expression of hsa-miR-425-5p
Log2 expression of DYNC1I2

Figure S7

Figure S7: Expression of hsa-miR425-5p and hsa-miR1915 in primary medulloblastoma samples and correlation of each candidate with *DYNC1I2* expression

(a) Violin plots show hsa-miR425-5p expression according to molecular subgroups of medulloblastoma (MB). (b) Quantification of hsa-miR-425-5p expression in the indicated brain tumor cell line models as determined by qRT-PCR. Data are presented as the mean ± SD of n = 4 independent experiments. (c+d) Correlation analysis of *DYNC1I2* expression and hsa-miR-425-5p (c) or hsa-miR-1915 (d) levels comparing FPKM expression values in 167 MB patient samples. (e) Subgroup specific correlation analysis of mRNA and miRNA sequencing data comparing FPKM expression values in 167 patient samples for *DYNC1I2* and hsa-miR-425-5p in non SHH MB (WNT, Group 3 and Group 4) and SHH MB tumors. Statistics were done by using Pearson correlation coefficient. Source data of gene expression via real time PCR are provided as a “Source Data file”.

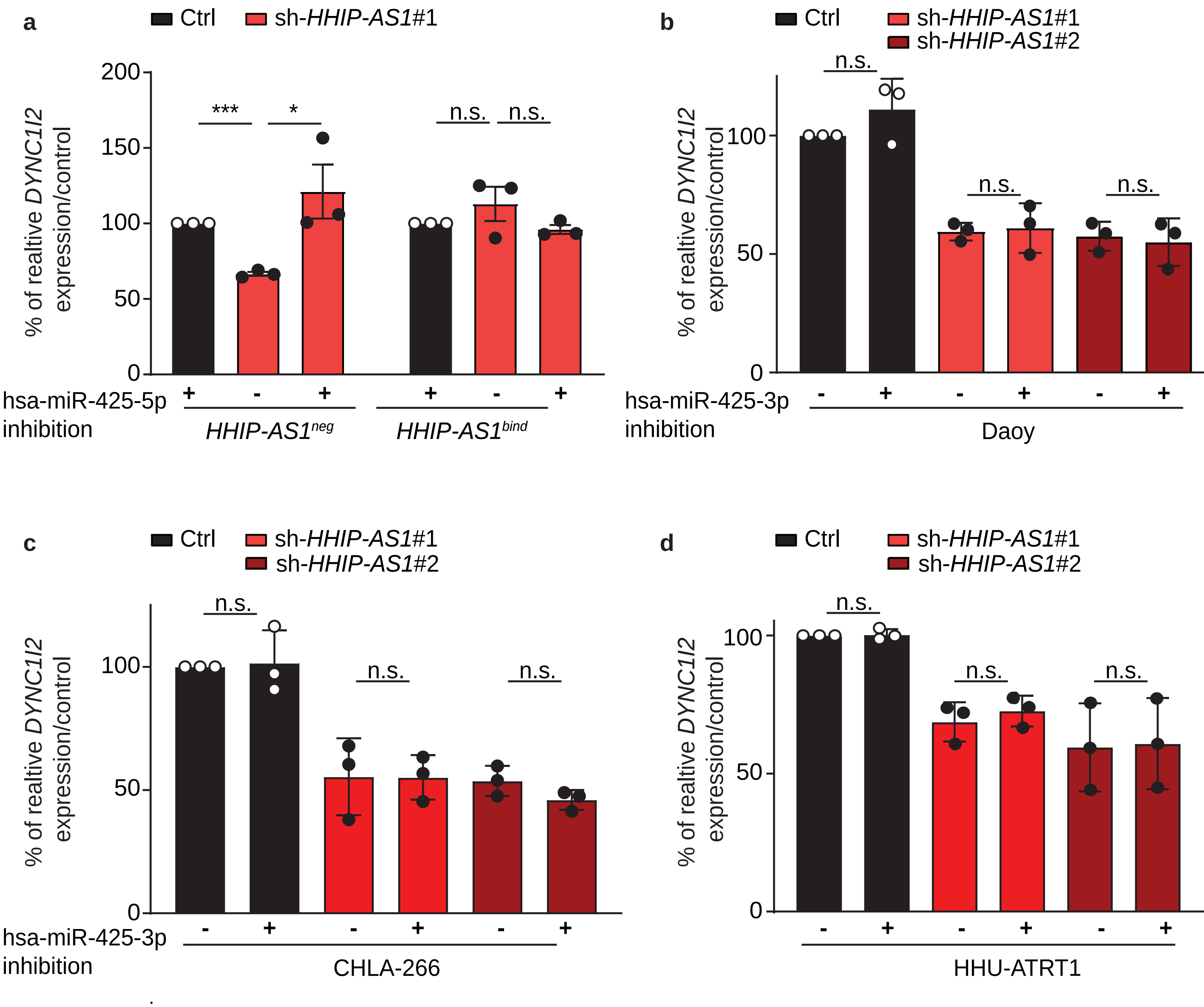
a
Ctrl
sh-HHIP-AS1#1
% of realtive DYNC1I2 expression/control
200
150
100
50
0
***
*
n.s.
n.s.
hsa-miR-425-5p inhibition
+ - + + - +
HHIP-AS1neg
HHIP-AS1bind
b
Ctrl
sh-HHIP-AS1#1
sh-HHIP-AS1#2
n.s.
100
50
0
hsa-miR-425-3p inhibition
- + - + - +
Daoy
c
Ctrl
sh-HHIP-AS1#1
sh-HHIP-AS1#2
n.s.
100
50
0
hsa-miR-425-3p inhibition
- + - + - +
CHLA-266
d
Ctrl
sh-HHIP-AS1#1
sh-HHIP-AS1#2
n.s.
100
50
0
- + - + - +
HHU-ATRT1

Figure S8

Figure S8 *HHIP-AS1* binding site blocks endogenous hsa-miR-425-5p function to maintain *DYNC1I2* levels

(a) Quantification of *DYNC1I2* expression level via qRT-PCR after *in vitro* transfection of stable *sh-HHIP-AS1* Daoy with *HHIP-AS1*^{bind} or *HHIP-AS1*^{neg} sequence. *HHIP-AS1*^{bind} induces higher expression of *DYNC1I2* mRNA, independent of hsa-miR-425-5p inhibition compared to a control condition where *HHIP-AS1*^{neg} was used. (b-d) *DYNC1I2* expression level was measured via qRT-PCR in Daoy (b), CHLA-266 (c) and HHU-ATRT1 (d) cells upon stable *HHIP-AS1* knockdown using two independent stable shRNAs (sh-*HHIP-AS1*#1 and sh-*HHIP-AS1*#2), in combination with or without transient inhibition of hsa-miR-425-3p. In panel (a), results are presented as the mean ± SEM and in panels b-d, results are presented as the mean ± SD of three independent experiments and expressed as percentage of *DYNC1I2* expression level. Statistical analysis was conducted by using Student's two-sided *t*-test; *p < 0.05, ***p < 0.001; n.s.= not significant. Source data and exact p-values are provided as a "Source Data file".

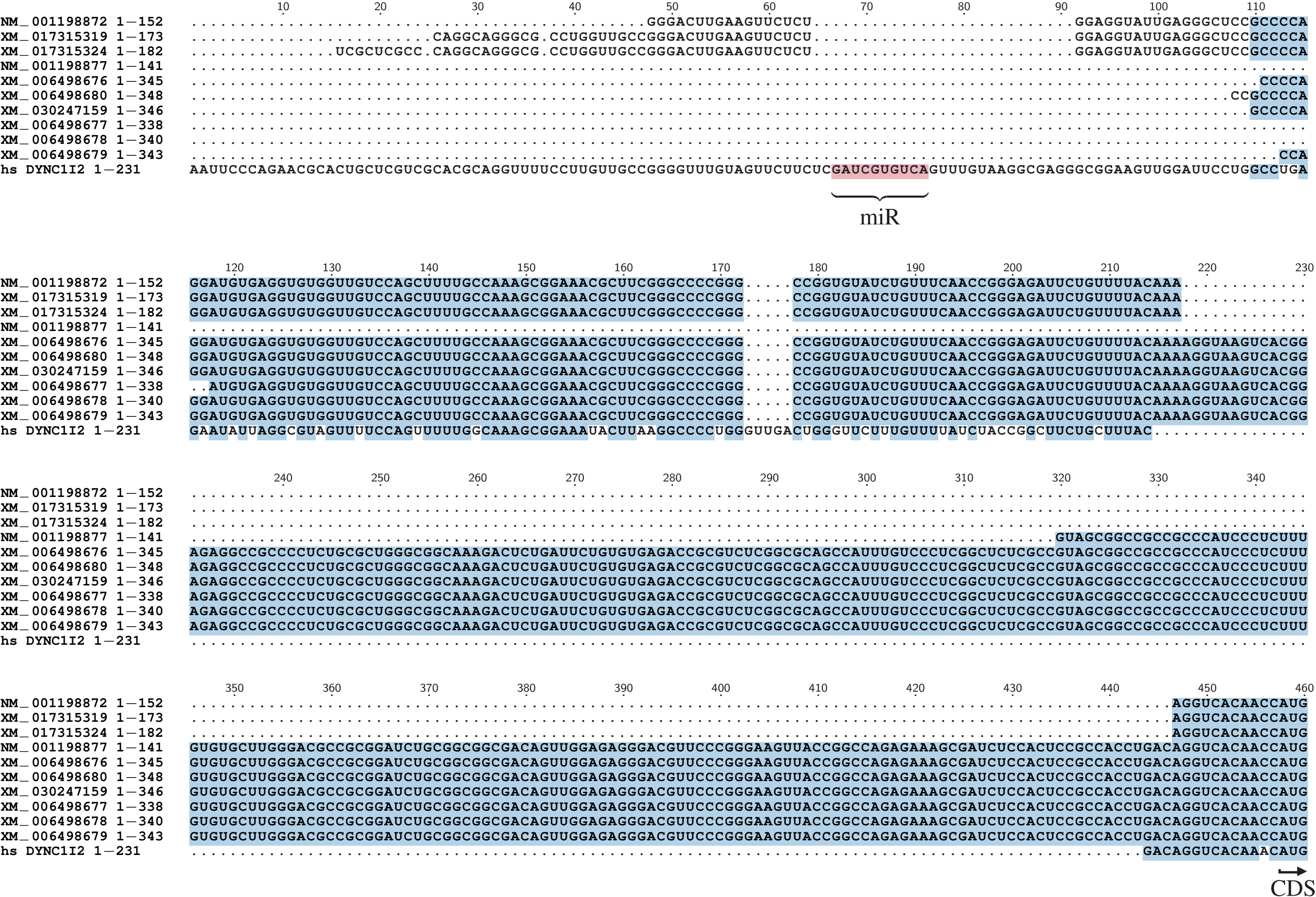
NM_001198872 1–152
XM_017315319 1–173
XM_017315324 1–182
NM_001198877 1–141
XM_006498676 1–345
XM_006498680 1–348
XM_030247159 1–346
XM_006498677 1–338
XM_006498678 1–340
XM_006498679 1–343
hs DYNC1I2 1–231
miR
CDS

Figure S9: Alignment of 5'-UTR regions of *dync1i2* from *mus musculus* and *DYNC1I2* from *homo sapiens*

In GenBank, 22 transcript variants of *dync1i2* are annotated in mouse chromosome 2 (GB LOCUS NC_000068); the following 5'-UTR regions of these variants were identical: NM_001198872 (nt 1--152), NM_001198873, NM_001198874, NM_001198875, and NM_001198876; XM_017315319 (nt 1--173) and XM_017315326; XM_017315324 (nt 1--182) and XM_030247161; NM_001198877 (nt 1--141), NM_001198878, NM_001347173, and NM_010064; XM_006498676 (nt 1--345) and_XM_030247156; XM_030247159 (nt 1--346) and XM_030247162; XM_006498679 (nt 1--343) and XM_030247151; the 5'-UTR regions of XM_006498680 (nt 1--348), XM_006498677 (nt 1--338), and XM_006498678 (nt 1--340) were unique. The sequences were aligned with the mafft method FFT-NS-i [2]; the alignment was drawn using TEXshade [3]. In the alignment, nucleotides identical in at least eight of the eleven sequences are shown on blue background; the binding position of hsa-miR-425-5p in hsDync1i2 (nt 67--76) is marked in red; the coding sequences (CDS) start at position 478 of the alignment.

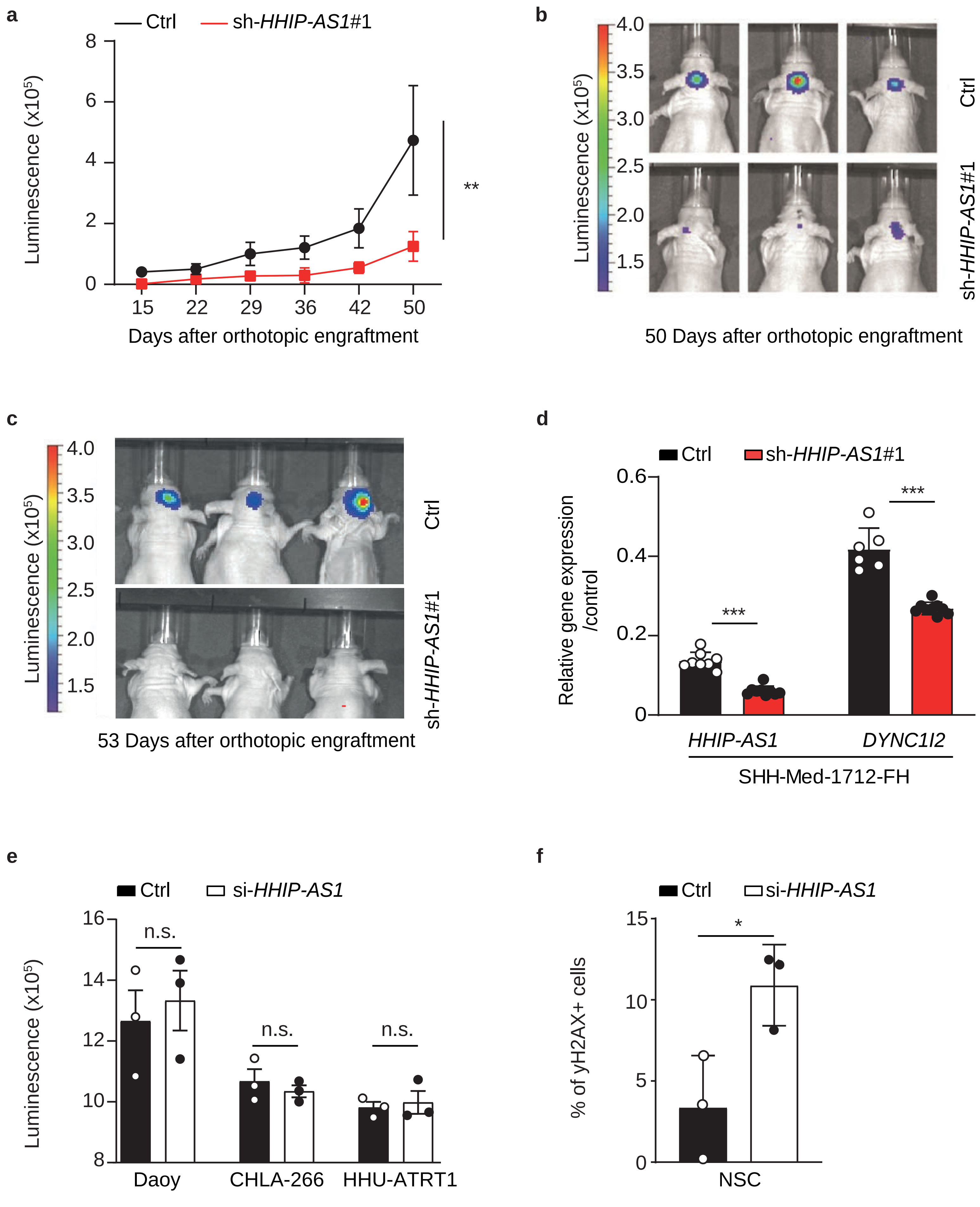

a
Ctrl
sh-HHIP-AS1#1
Luminescence (x10^5)
8
6
4
2
0
15
22
29
36
42
50
Days after orthotopic engraftment
**
b
Luminescence (x10^5)
4.0
3.5
3.0
2.5
2.0
1.5
Ctrl
sh-HHIP-AS1#1
50 Days after orthotopic engraftment
c
Luminescence (x10^5)
4.0
3.5
3.0
2.5
2.0
1.5
Ctrl
sh-HHIP-AS1#1
53 Days after orthotopic engraftment
d
Ctrl
sh-HHIP-AS1#1
Relative gene expression /control
0.6
0.4
0.2
0
***
***
HHIP-AS1
DYNC1I2
SHH-Med-1712-FH
e
Ctrl
si-HHIP-AS1
Luminescence (x10^5)
16
14
12
10
8
n.s.
n.s.
n.s.
Daoy
CHLA-266
HHU-ATRT1
f
Ctrl
si-HHIP-AS1
% of γH2AX+ cells
15
10
5
0
*
NSC

Figure S10

Figure S10: *In vivo* validation of *HHIP-AS1* pro-tumorigenic effect in SHH-driven brain tumor models

(a) Growth tumor curve, measured by luminescence, of nude mice orthotopically engrafted with stably *HHIP-AS1*-silenced (sh-*HHIP-AS1*#1) Daoy cells compared to corresponding control mice (Ctrl) engrafted with sh-scramble infected cells. Values are presented as the mean ± SEM and statistical analysis was done using ANOVA of n ≥ 6 animals per condition; **p < 0.01. (b+c) Representative bio-luminescent optical imaging in tumor-bearing mice (in panel (b) Daoy and in (c) CHLA-266). Images are composed of both photographic and luminescent overlay. Top row: control, lower row: *HHIP-AS1*-silenced (sh-*HHIP-AS1*#1). (d) Bar graph depicting *DYNC1I2* and *HHIP-AS1* expression as measured by qRT-PCR after transient knockdown of *HHIP-AS1* (sh-*HHIP-AS1#1*) normalized to control (sh-scr) in SHH-Med-1712-FH PDX tumor tissue. Values are presented as the mean ± SD and statistical analysis was done by Student's two-sided *t*-test, ***p < 0.001 of n ≥ 8 mice brain tissue samples. (e) Caspase activity measured via caspase-Glo 3/7 assay system in Daoy, CHLA-266 and HHU-ATRT cells with transient knockdown of *HHIP-AS1* compared to corresponding controls (Ctrl), respectively. Bar graphs are presented as the mean ± SEM of three independent experiments; statistical analysis was done by Student's *t*-test; n.s.= not significant. (f) DNA damage after transient *HHIP-AS1* depletion in neuronal stem cells (NSC) was measured using immunofluorescence of γH2AX (red) and the percentage of positive stained cells is plotted in the bar graphs as mean ± SD of three independent experiments. Statistical analysis was done by Student's *t*-test, *p < 0.05. Source data and exact p-values are provided as a "Source Data file".

a

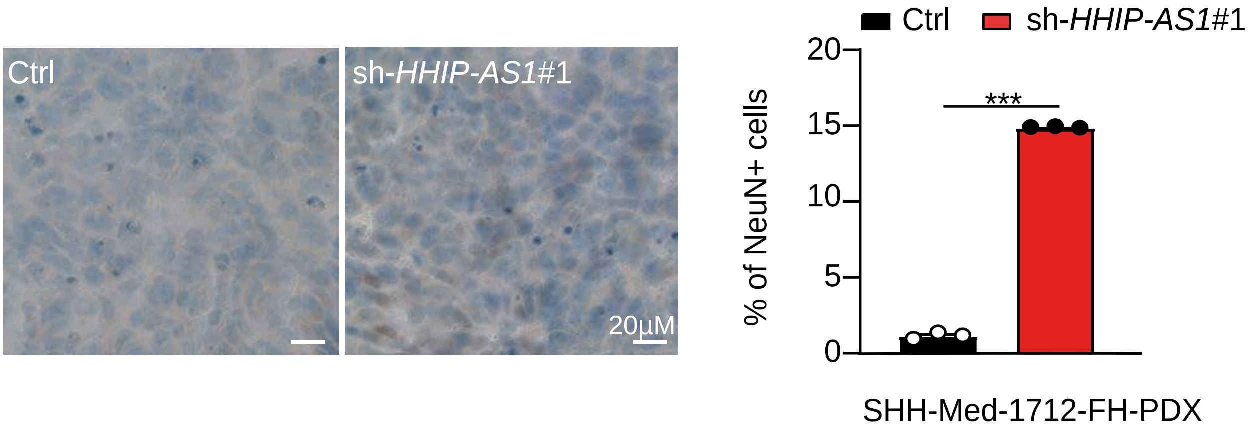

b

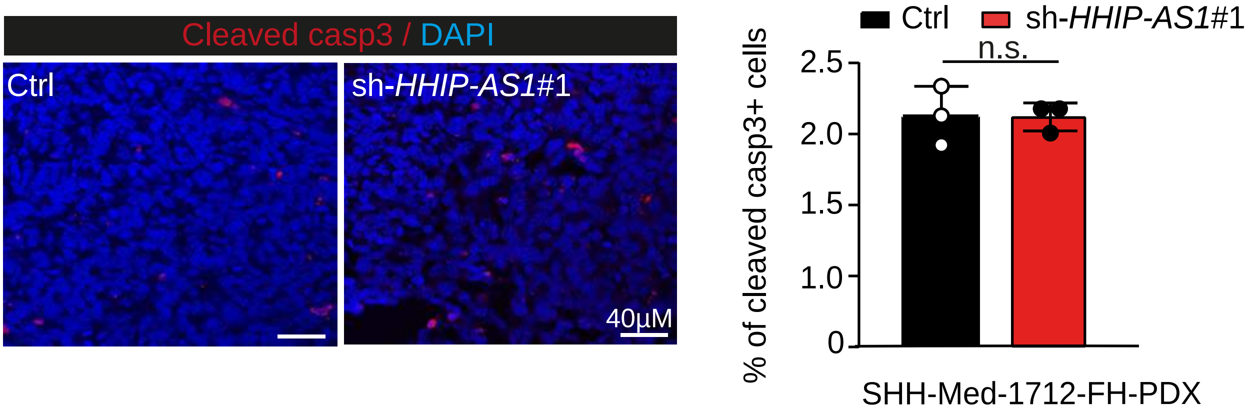

Figure S11

Figure S11: Immunostaining of tumor tissue from SHH-driven brain tumor models

Three controls and three *HHIP-AS1*-depleted SHH-Med-1712-FH tumors were either DAB stained for NeuN, white scale bar: 20µm, (a) or immunofluorescent stained for cleaved caspase 3, white scale bar: 40µm (b) and the percentage of positive stained tumor cells is plotted in the bar graphs as mean ± SD. Nuclei are stained with either Hematoxylin or DAPI (blue). Both, control (ctrl = sh-*scr*) and sh-*HHIP-AS1*#1 tumor tissues, are shown in the two representative images. Statistical analysis was done by Student's two-sided *t*-test, ***p< 0.001; n.s.= not significant. Source data and exact p-values are provided as a "Source Data file".

Table S1: Computationally derived pairing sequence of lncRNA with mRNA

Interaction between ENST00000512359(ncRNA) and ENST00000534253(mRNA)

	**Genomic coordinates of transcripts**	**Interaction energy bewteen these transcripts**	**Location of the interaction site on mRNA**
ENST00000512359(ncRNA)	chr4:145564525-145582509	SumEnergy -391.48 kcal/mol, MinEnergy -19.59 kcal/mol	5´UTR
ENST00000534253(mRNA)	chr2:172543980-172604919		

	**Binding site structure predicted by IntaRNA**	**Joint secondary structure predicted by RactIP**
ENST00000512359(ncRNA)	CTTCTCGATCGTGTCAGTTTGTAAGGCGAGGGCGGAAGTTG	CTTCTCGATCGTGTCAGTTTGTAAGGCGAGGGCGGAAGTTG
	...........((((((((((..(((((((((.........	..((.......(((((((((((.(((((((((.........
ENST00000534253(mRNA)	TTGCAACCCTTGCCTACAACCAGACTGACAGCATAATTTCTTAGGAATCA	TTGCAACCCTTGCCTACAACCAGACTGACAGCATAATTTCTTAGGAATCA
	......))))))))).....)))))))))))....................	......))))))))))....))))))))))..............))....

Table S2: miRNA binding sites within the predicted interaction region

miRNA	Target	Total Score	Total Energy	Max Score	Max Energy	Strand	Len1	Len2	Positions	Number hits
hsa-miR-425-5p	DYNC1I2_bind	140.00	-16.16	140.00	-16.16	382	23	31	1	1
hsa-miR-888-3p	DYNC1I2_bind	145.00	-12.53	145.00	-12.53	780	22	31	1	1
hsa-miR-1915-5p	DYNC1I2_bind	143.00	-21.36	143.00	-21.36	998	22	31	5	1
hsa-miR-718	DYNC1I2_bind	157.00	-28.14	157.00	-28.14	1025	21	31	11	1
hsa-miR-6730-3p	DYNC1I2_bind	140.00	-9.60	140.00	-9.60	2139	22	31	1	1
hsa-miR-6803-3p	DYNC1I2_bind	157.00	-22.33	157.00	-22.33	2284	22	31	6	1

Supplementary Table S3: Oligonucleotide sequences used in this study:

**Oligonucleotides sequences**	
Scramble *scr* (sh-*scr*)	fw-5´CCGGCCTAAGGTTAAGTCGCCCTCGCTCGAGCGAGGGCGACTTAACCTTAGGTTTTTG3´
cloned in pLK0.1-TRC-Puro or in pLVMTH for *in vivo* experiments	rv-5´AATTCAAAAACCTAAGGTTAAGTCGCCCTCGCTCGAGCGAGGGCGACTTAACCTTAGG3’
sh-*HHIP-AS1* #1	fw-5´CCGGAGAGGATAGCCATGCATACCTCGAGGTATGCATGGCTATCCTCTTTTTG3´
cloned in pLK0.1-TRC-Puro or in pLVMTH for *in vivo* experiments	rv-5´AATTCAAAAAGAGGATAGCCATGCATACCTCGAGGTATGCATGGCTATCCTCT3´
sh-*HHIP-AS1* #2	fw-5´CCGGGAGGATAGCCATGCATACACTCGAGTGTATGCATGGCTATCCTCTTTTTG3´
cloned in pLK0.1-TRC-Puro	rv-5´AATTCAAAAAGAGGATAGCCATGCATACACTCGAGTGTATGCATGGCTATCCTC3´
HHIP-AS1 ^{bind} “positive binding” for RNA-RNA interaction (*DYNC1I2* mRNA stability experiments and Bio-Layer interferometry)	5´CCCTTGCCTACAACCAGACTGACA3´

HHIP-AS1 ^{neg}<br>“negative binding” for RNA-RNA interaction (*DYNC1I2* mRNA stability experiments and Bio-Layer interferometry)	5´TTCAGCCTCCAAGGGGGCTTTTAA3´
gDYNC OE	fw-5´caccGAGTGGGAAATCTAAAGGAG3´<br><br>rv-5´aaacCTCCTTTAGATTTCCCACTC3´
gGLI OE	OE#1<br><br>fw-5´caccgAGGCCCCGACAACCAGATTG3´<br><br>rv-5´aaacCAATCTGGTTGTCGGGGCCT3´<br><br>OE#2<br><br>fw-5´caccgAGATTGAGGAAAATATTGCG3´<br><br>rv-5´aaacCGCAATATTTTCCTCAATCT3´
g*HHIP-AS1* OE	fw-5´caccGCGAGAAGCGGTGACGTCAA3´<br><br>rv-5´aaacTTGACGTCACCGCTTCTCGC3´

**Purchased Oligonucleotides**	**Company**	**Catalogue-number**
singleplex miRNA Assays hsa-miR425-5p	Thermo Fisher Scientific	#SM-10204
Syn miRNA inhib hum hsa-miR-425-3p	Merck	# HSTUD0568
singleplex miRNA Assays hsa-miR191	Thermo Fisher Scientific	#SM-20786
pre-miR miRNA Precursor Negative Control #1	Thermo Fisher Scientific	#AM17110
pre-miR miRNA Precursor hsa-miR-425-5p	Thermo Fisher Scientific	#17100
si-*negative* -POOL	siTOOLs Biotech	#Negative control siPOOL 5
siRNA pools *HHIP-AS1*	siTOOLs Biotech	#646576
siRNA pools *DYNC1I2*	siTOOLs Biotech	#1781
siRNA pools *GLI1*	siTOOLs Biotech	#2735
siRNA pools *GLI2*	siTOOLs Biotech	#2736
raPOOL *HHIP-AS1*	siTOOLs Biotech	#raPOOL646576

References Supplementary Info

1 Fickett, J. W., Torney, D. C. & Wolf, D. R. Base compositional structure of genomes. *Genomics* **13**, 1056-1064, doi:10.1016/0888-7543(92)90019-o (1992).

2 Katoh, K. & Toh, H. Recent developments in the MAFFT multiple sequence alignment program. *Brief Bioinform* **9**, 286-298, doi:10.1093/bib/bbn013 (2008).

3 Beitz, E. TEXshade: shading and labeling of multiple sequence alignments using LATEX2 epsilon. *Bioinformatics* **16**, 135-139, doi:10.1093/bioinformatics/16.2.135 (2000).
