# Supplementary material for: The HHIP-AS1 lncRNA promotes tumorigenicity through stabilization of dynein complex 1 in human SHH-driven tumors
Source: Nat Commun. 2022 Jul 13;13:4061. doi: 10.1038/s41467-022-31574-z (PMC9279496; doi:10.1038/s41467-022-31574-z)
Supplement: Supplementary file 2 — Description of Additional Supplementary Files [file 41467_2022_31574_MOESM2_ESM.pdf]

Description of Additional Supplementary Files

File Name: Supplementary Data 1

Description: **Differentially expressed transcripts in RNA sequencing data in medulloblastoma** Differentially expressed transcripts in RNA sequencing data by comparing sonic hedgehog (SHH) MB (n = 58) to non SHH MB subgroup samples (n = 164) (Platform, R2). The approach confirmed several known protein-coding SHH mediators including GLI1, GLI2, HHIP and Atonal BHLH Transcription Factor 1, and revealed lncRNAs deregulated in SHH MB. MB= medulloblastoma, HHIP= hedgehog interacting protein, GLI= Zinc finger protein. Statistical analysis was performed using one-way ANOVA with post-hoc Tukey HSD.
