# Supplementary material for: The HHIP-AS1 lncRNA promotes tumorigenicity through stabilization of dynein complex 1 in human SHH-driven tumors
Source: Nat Commun. 2022 Jul 13;13:4061. doi: 10.1038/s41467-022-31574-z (PMC9279496; doi:10.1038/s41467-022-31574-z)
Supplement: Supplementary file 4 — Reporting Summary [file 41467_2022_31574_MOESM4_ESM.pdf]

nature research

Corresponding author(s): Jasmin Bartl, Olivier Ayrault, Marc Remke

Last updated by author(s): Jun 2, 2022

Reporting Summary

Nature Research wishes to improve the reproducibility of the work that we publish. This form provides structure for consistency and transparency in reporting. For further information on Nature Research policies, see our Editorial Policies and the Editorial Policy Checklist.

Statistics

For all statistical analyses, confirm that the following items are present in the figure legend, table legend, main text, or Methods section.

n/a	Confirmed	
☐	☒	The exact sample size (*n*) for each experimental group/condition, given as a discrete number and unit of measurement
☐	☒	A statement on whether measurements were taken from distinct samples or whether the same sample was measured repeatedly
☐	☒	The statistical test(s) used AND whether they are one- or two-sided<br>*Only common tests should be described solely by name; describe more complex techniques in the Methods section.*
☒	☐	A description of all covariates tested
☒	☐	A description of any assumptions or corrections, such as tests of normality and adjustment for multiple comparisons
☐	☒	A full description of the statistical parameters including central tendency (e.g. means) or other basic estimates (e.g. regression coefficient) AND variation (e.g. standard deviation) or associated estimates of uncertainty (e.g. confidence intervals)
☐	☒	For null hypothesis testing, the test statistic (e.g. *F*, *t*, *r*) with confidence intervals, effect sizes, degrees of freedom and *P* value noted<br>*Give P values as exact values whenever suitable.*
☒	☐	For Bayesian analysis, information on the choice of priors and Markov chain Monte Carlo settings
☒	☐	For hierarchical and complex designs, identification of the appropriate level for tests and full reporting of outcomes
☐	☒	Estimates of effect sizes (e.g. Cohen's *d*, Pearson's *r*), indicating how they were calculated

Our web collection on statistics for biologists contains articles on many of the points above.

Software and code

Policy information about availability of computer code

Data collection

PRIDE database with the #PXD016550 accession code
GenBank with the #GSE140741 accession code
UniProt database (human) https://www.uniprot.org/

Data analysis

Partek® Flow® software, version 5.0 Partek Inc., St. Louis, MO, USA. http://www.partek.com/
GraphPad PRISM ® Version 9 Graphpad Software, Inc https://www.graphpad.com/scientific-software/prism/
IntaRNA http://rna.informatik.unifreiburg.de/IntaRNA/Input.jsp
Bioinformatics Web Server for RNA http://rtools.cbrc.jp
RNAFOLD "ViennaRNA package" v. 2.4.6
STAR Version 2.4.1d https://github.com/alexdobin/STAR/releases
BOWTIE2 Version 2.2.5 http://bowtie-bio.sourceforge.net/bowtie2/news.shtml
Proteome Discoverer Version 1.4.1.14 Thermo Fisher Scientific
Mascot Version 2.4 Matrix Science
Progenesis QI for Proteomics Version 2.0, Nonlinear Dynamics Waters Corporation
MAFFT version 7
Multiple alignment program for amino acid or nucleotide sequences https://mafft.cbrc.jp/alignment/server/
Image J 1.53q, Java 1.8.0_322 https://imagej.nih.gov/ij/download.html
ECR browser https://ecrbrowser.dcode.org/
R2 R2: Genomics Analysis and Visualization Platform http://r2.amc.nl
Ensembl release 84 https://www.ensembl.org/index.html

For manuscripts utilizing custom algorithms or software that are central to the research but not yet described in published literature, software must be made available to editors and reviewers. We strongly encourage code deposition in a community repository (e.g. GitHub). See the Nature Research guidelines for submitting code & software for further information.

Data

Policy information about availability of data

All manuscripts must include a data availability statement. This statement should provide the following information, where applicable:

- Accession codes, unique identifiers, or web links for publicly available datasets
- A list of figures that have associated raw data
- A description of any restrictions on data availability

RNA sequence data that support the findings of this study have been deposited in GenBank with the #GSE140741 accession code and proteomic data have been deposited in ProteomeXchange PRIDE database with the #PXD016550 accession code. The raw data behind data points in figures and that support the findings of this study are available in the linked source file table. The authors declare that the data supporting the findings of this study are available within the paper and its supplementary information files.

Field-specific reporting

Please select the one below that is the best fit for your research. If you are not sure, read the appropriate sections before making your selection.

☒ Life sciences ☐ Behavioural & social sciences ☐ Ecological, evolutionary & environmental sciences

For a reference copy of the document with all sections, see nature.com/documents/nr-reporting-summary-flat.pdf

Life sciences study design

All studies must disclose on these points even when the disclosure is negative.

Sample size	No statistical methods were used to predetermine the experimental sample size. Sample sizes are indicated in the figure legends. Sample size was determined based on preliminary experiments that defined the adequate number of samples to consistently identify differences between groups (e.g. knockdown versus control, treatment vs non treated cells, ect)
Data exclusions	no data were excluded
Replication	at least three independent (different day, different passage of cells, etc) experiments were performed and replicates were successfull
Randomization	we tried to ensure that the control sample is not always the first one and the knockdown or treated sample is not always the last one to be processed. We had no fixed sequence for perfoming the experiments. We also treid to avoid 'plate-effect'. In general, the layout of samples within and across plates were randomly arranged so that valid inferences can be obtained even if these artifacts are present. Additionally, all experiments were done on different days.
Blinding	collection of data (performing experiments) and data analysis/intepretation was quite often done from different persons. Example: EdU proliferation staining was done by Sarah Göbbels, technical assistent. Calculation of data for proliferation was done by Dr Jasmin Bartl. See also author contribution for further details.

Reporting for specific materials, systems and methods

We require information from authors about some types of materials, experimental systems and methods used in many studies. Here, indicate whether each material, system or method listed is relevant to your study. If you are not sure if a list item applies to your research, read the appropriate section before selecting a response.

Materials & experimental systems

n/a	Involved in the study	
☐	☒	Antibodies
☐	☒	Eukaryotic cell lines
☒	☐	Palaeontology and archaeology
☐	☒	Animals and other organisms
☐	☒	Human research participants
☒	☐	Clinical data
☒	☐	Dual use research of concern

Methods

n/a	Involved in the study	
☐	☒	ChIP-seq
☒	☐	Flow cytometry
☒	☐	MRI-based neuroimaging

Antibodies

Antibodies used	Mouse anti-beta-actin from Santa Cruz #sc-47778: RRID: AB_2714189<br>Mouse anti-GLI2 from Santa Cruz #sc-271786 RRID: AB_10708124<br>Rabbit anti-GLI1 from Cell Signaling #V812: RRID:AB_2294745<br>Rabbit anti-DYNC1I2 from Atlas Antibodies #HPA040619 RRID:AB_2677045

	Mouse anti-HHIP from Abnova #H00064399-M01 RRID:AB_518852<br>Mouse anti-β-Actin from Cell Signaling #3700 RRID:AB_2242334<br>Rabbit anti-Ki67 from Millipore & Leica #AB9260 RRID:AB_2142366<br>Rat anti-BrdU from AbDserotec #OBT0030G RRID:AB_609567<br>Rabbit anti-Pericentrin from Abcam #ab4448 RRID:AB_304461<br>Mouse anti-Acetylated tubulin from Merck #T6793 RRID:AB_477585<br>Chicken anti-Rabbit IgG (H+L) cross-adsorbed secondary antibody, Alexa Fluor 488 labeled from Thermo Fisher Scientific #A-21441 RRID:AB_141735<br>Goat anti-Mouse IgG2b cross-adsorbed secondary antibody, Alexa Fluor 594 labeled Thermo Fisher Scientific from #A-21145 RRID:AB_1500892<br>mouse anti-a-tubulin from Santa Cruz #sc-8035 RRID:AB_628408<br>rabbit anti-NeuN from Abcam, #ab177487 RRID:AB_2532109<br>rabbit anti-phospho-histone H2A.X from Cell Signaling #9718 RRID:AB_2118009<br>rabbit anti-Cleaved Caspase-3 from Cell Signaling #9661S RRID:AB_2341188
Validation	All used antibody are registrated in RRID and can be finde in "The Antibody Registry":<br>https://antibodyregistry.org/search.php?q=AB_2714189<br>https://antibodyregistry.org/search.php?q=AB_10708124<br>https://antibodyregistry.org/search.php?q=AB_2294745<br>https://antibodyregistry.org/search.php?q=AB_2677045<br>https://antibodyregistry.org/search.php?q=AB_518852<br>http://antibodyregistry.org/search.php?q=AB_2242334<br>https://antibodyregistry.org/search.php?q=AB_2142366<br>https://antibodyregistry.org/search.php?q=AB_609567<br>https://antibodyregistry.org/search.php?q=AB_304461<br>https://antibodyregistry.org/search.php?q=AB_477585<br>https://antibodyregistry.org/search.php?q=AB_141735<br>https://antibodyregistry.org/search.php?q=AB_1500892<br>https://antibodyregistry.org/search?q=sc-8035<br>https://antibodyregistry.org/search?q=ab177487<br>https://antibodyregistry.org/search?q=9718<br>https://antibodyregistry.org/search?q=9661S

Eukaryotic cell lines

Policy information about cell lines

Cell line source(s)	Human: Daoy ATCC #HTB-186 RRID:CVCL_1167<br>Human: CHLA-266 CCcells RRID:CVCL_M149<br>Human: HHU-ATRT1 Generated at the University Hospital Düsseldorf<br>Human: HD-MB03 Gift of Dr. Till Milde, KiTZ, Hopp Children´s Cancer Center Heidelberg, generated in Heidelberg<br>Human: HEK293T Gift of Dr. Ute Fischer, University Hospital Düsseldorf (original ATCC: #CRL-1573 RRID:CVCL_0045)<br>Human: UI226 Gift of Dr Henry Kuonen, University Stanford, generated in Stanford<br>Human: RH30 Gift of Prof Simone Fulda, University of Kiel (original ATCC #CRL-2061 RRID:CVCL_0041)<br>Human: NSC purchased from Thermo Fischer Scientific #GIBCO N7800100 RRID: CVCL_U176<br>Human: CHLA-01 purchased from ATCC #CRL-3021 RRID: CVCL_B044<br>Human: CHLA-04 purchased from ATCC #CRL-3036 RRID: CVCL_0F38
Authentication	all recieved and purchased cell lines were STR profiled
Mycoplasma contamination	all used cell lines were mycoplasmen tested and had no mycoplasmen contamination
Commonly misidentified lines (See ICLAC register)	none

Animals and other organisms

Policy information about studies involving animals; ARRIVE guidelines recommended for reporting animal research

Laboratory animals	For the orthotopic brain tumor models we used female NMRI-Foxn1nu/nu (8 weeks old) that were purchased from Janvier Labs, Le Genest-Saint-Isle, France. Other animal models were Med-1712-FH (SHH MB PDX) from Olson lab and ICN-MB12 (SHH MB PDX) from Ayrault lab. Both PDXs were maintained in female NMRI-Foxn1nu/nu (8 weeks old). Temperature (20°C–22°C) and humidity (40%–50%) on a 12-hour light/12-hour dark cycle.
Wild animals	none
Field-collected samples	none
Ethics oversight	We followed both European and national regulations for animal housing, care and experimentation (Directive 86/609). The use of

Ethics oversight | animals was approved by the reporting ethical committee (CCEA-IC, Institut Curie,) and the ministry under the agreement #03130.20.

Note that full information on the approval of the study protocol must also be provided in the manuscript.

Human research participants

Policy information about studies involving human research participants

Population characteristics: Patient-derived MB cells were freshly isolated from tissue samples obtained by surgical resection at the Department of Neurosurgery, University Hospital Düsseldorf. n=2 SHH-MB patients.

Recruitment: *Describe how participants were recruited. Outline any potential self-selection bias or other biases that may be present and how these are likely to impact results.*

Ethics oversight: Informed consent by the patients and approval by the institutional review board (study number: 2018-102).

Note that full information on the approval of the study protocol must also be provided in the manuscript.

ChIP-seq

Data deposition

☒ Confirm that both raw and final processed data have been deposited in a public database such as GEO.

☒ Confirm that you have deposited or provided access to graph files (e.g. BED files) for the called peaks.

Data access links
May remain private before publication.
Data were re-analyzed from a previously published publication: Lin, et al "Active medulloblastoma enhancers reveal subgroup-specific cellular origins" Nature 2016. https://pubmed.ncbi.nlm.nih.gov/26814967/
No new data were generated for the submitted publication

Files in database submission: Data were re-analyzed from a previously published publication: Lin, et al "Active medulloblastoma enhancers reveal subgroup-specific cellular origins" Nature 2016. No new data were generated for the submitted publication

Genome browser session (e.g. UCSC): Data were re-analyzed from a previously published publication: Lin, et al "Active medulloblastoma enhancers reveal subgroup-specific cellular origins" Nature 2016. No new data were generated for the submitted publication

Methodology

Replicates: see description in previously published publication: Lin, et al "Active medulloblastoma enhancers reveal subgroup-specific cellular origins" Nature 2016. No new data were generated for the submitted publication Bartl, et al.

Sequencing depth: see description in previously published publication: Lin, et al "Active medulloblastoma enhancers reveal subgroup-specific cellular origins" Nature 2016. No new data were generated for the submitted publication Bartl, et al.

Antibodies: see description in previously published publication: Lin, et al "Active medulloblastoma enhancers reveal subgroup-specific cellular origins" Nature 2016. No new data were generated for the submitted publication Bartl, et al.

Peak calling parameters: see description in previously published publication: Lin, et al "Active medulloblastoma enhancers reveal subgroup-specific cellular origins" Nature 2016. No new data were generated for the submitted publication Bartl, et al.

Data quality: see description in previously published publication: Lin, et al "Active medulloblastoma enhancers reveal subgroup-specific cellular origins" Nature 2016. No new data were generated for the submitted publication Bartl, et al.

Software: see description in previously published publication: Lin, et al "Active medulloblastoma enhancers reveal subgroup-specific cellular origins" Nature 2016. No new data were generated for the submitted publication Bartl, et al.
